# Antimalarial Potential of Heme-Targeting Dimeric Compounds: Binding Efficacy vs. Membrane Retention Effects

**DOI:** 10.1021/acsomega.5c06934

**Published:** 2026-01-08

**Authors:** Victor Matheus Kemmer, Fabricio Santos, Fernanda Alice de Oliveira, Ana Claudia de Sousa Pinto, Amanda Luisa da Fonseca, Letícia Aparecida da Silva, Helen Gonçalves Marques, Fabio Vieira dos Santos, Cleber Paulo Andrada Anconi, Franco Henrique Andrade Leite, David Bacelar Costa, Fernando de Pilla Varotti, Clébio Soares Nascimento, Luciana Guimarães, Gustavo Henrique Ribeiro Viana, Renato Márcio Ribeiro-Viana, Anna Paola Butera

**Affiliations:** † Departamento de Química, 37894 Universidade Estadual de Londrina, Londrina 86057-970, Brazil; ‡ Núcleo de Pesquisa em Química Biológica (NQBio), 74383 Universidade Federal de São João del Rei, Divinópolis 35501-296, Brazil; § Laboratório de Bioquímica Medicinal, 573437 Universidade Federal de São João del Rei, Divinópolis 35501-296, Brazil; ∥ Departamento de Ciências Naturais, 74383 Universidade Federal de São João del Rei, Campus Dom Bosco, São João Del Rei 36301-160, Brazil; ⊥ Laboratório de Biologia Celular e Mutagênese, Universidade Federal de São João del Rei, Divinópolis 35501-296, Brazil; # Departamento de Química, Instituto de Ciências Naturais, 67739 Universidade Federal de Lavras, Lavras 37200-900, Brazil; ∇ Laboratório de Químioinformática e Avaliação Biológica, 67836 Universidade Estadual de Feira de Santana, Feira de Santana 44036-900, Brazil; ○ Programa de Pós-graduação em Ciência e Engenharia de Materiais, 541505 Universidade Tecnológica Federal do Paraná, Londrina 86036-370, Brazil

## Abstract

Malaria, caused by Plasmodium parasites, remains a major global health burden with high morbidity and mortality rates. Despite the availability of treatments, the therapeutic arsenal is limited, and drug resistance poses a significant challenge. Thus, discovering new antimalarial compounds is critical, and understanding their mechanisms of action is key. One well-studied target is the heme group, which plays a central role in the degradation of the parasite’s hemoglobin and infection success. This study describes the dimerization of 3-alkylpyridine derivatives, a class of compounds known to interact with heme, with the aim of enhancing their antimalarial activity. The dimers were analyzed for heme-binding affinity via UV–vis spectroscopy (*K*_d_ determination) and further investigated through semiempirical GFN2-xTB and with the B97–3c functional with double (def2-SVP) and triple (def2-TZVP) zeta basis set quality, confirming effective interaction. However, antimalarial assays revealed modest IC50 values (46–140 μM). In silico membrane permeation studies showed that the compounds penetrate lipid bilayers effectively. However, they remain trapped within the membrane for a long time, which may reduce their intracellular availability and limit their efficacy. Although these compounds exhibit strong target binding, their antimalarial activity is hindered by unfavorable pharmacokinetic properties. These findings emphasize the importance of physicochemical properties in antimalarial drug development and suggest that advanced drug delivery systems could overcome these limitations in future studies.

## Graphical abstract

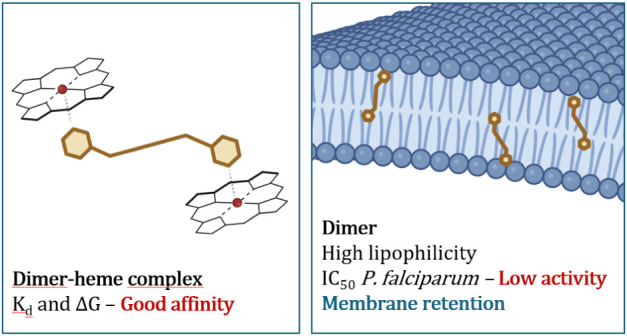

## Introduction

*Plasmodium falciparum* infections are responsible for the greatest morbidity and mortality rates, making malaria a significant global health issue.^1^ Despite the effectiveness of artemisinin-based combination therapies (ACTs), the emergence of resistant parasite strains poses a significant challenge to current treatment strategies in malaria.^2^ Determining the mechanism of action (MoA) of antimalarial drugs remains a significant challenge in malaria research, despite recent advancements in molecular and proteomic technology.^3–5^ Understanding the precise MoA is crucial for optimizing existing therapies and developing new drugs to combat the evolving threat of drug-resistant malaria.^6^ One of the primary difficulties is the reliance on phenotypic assays to identify new antimalarial compounds. While effective in discovering compounds with antiparasitic activity, these screens do not inherently provide information about the specific molecular targets involved. Further studies, such as those using in silico approaches, are required to uncover the underlying mechanisms.^7^

Consequently, the search for novel antimalarial compounds with distinct MoA is imperative. In this context, dimeric molecules have gained attention because of their enhanced bioactivity and potential to circumvent resistance mechanisms.^8–10^

The rational design of dimeric antimalarial agents is based on the hypothesis that linking two pharmacophores can improve molecular interactions with key biological targets. Heme metabolism remains an attractive target because Plasmodium parasites rely on hemoglobin degradation, leading to the release of toxic free heme that must be detoxified into hemozoin. Interference with this process has been a successful strategy for several antimalarial drugs, including chloroquine and artemisinin derivatives.^11–13^

Among the promising structural classes investigated for antimalarial activity, 3-alkylpyridine marine alkaloids (3-APA) have shown significant potential.^14–17^ These compounds have been explored for their ability to interfere with heme detoxification pathways, forming stable complexes with ferriprotoporphyrin IX, thereby disrupting hemozoin formation. The structural versatility of 3-APA alkaloids allows for the rational modification of their molecular framework, including dimerization strategies aimed at enhancing their pharmacological properties. Given their promising biological activity, dimeric analogs of 3-APA alkaloids represent an attractive scaffold for the development of next-generation antimalarial agents.

Given the potent antiplasmodial activity of 3-APA derivatives, this study focused on the dimerization of these compounds and the evaluation of their antiplasmodial activity and cytotoxicity, as well as both in silico and in vitro analyses. By exploring these aspects, we aim to provide valuable insights into the potential of dimeric 3-APA derivatives as novel therapeutic candidates for malaria.

## Results and Discussion

Given that the dimerization of the 3-pyridinopropoxy portion may favor the formation of a 2:1 complex, whereby the dimers could potentially inhibit the formation of hemozoin and, consequently, exhibit antiplasmodial activity, dimers **1**-**4** were proposed (Figure 1A). Generally, their structures consist of two 3-pyridinopropoxy units (red, in Figure 1) spaced by a linear aliphatic chain. Dimers **1** (18 atoms between the oxigens in red of 3-APA) and **2** feature a succinic group (in blue) as the central spacer (4 atoms between the oxigens in red of 3-APA). Additionally, dimer **1** includes an intermediate spacer chain of six carbons (in black), which is expected to maintain a greater distance between the 3-pyridinopropoxy units (18 atoms) compared to the other series members. Among the four proposed dimers, dimer **1** is most structurally similar to the compounds from the precursor study (Figure 1B). It retains the oxygen of the 3-pyridinopropoxy unit, forming an ether bond with an alkyl chain. Thus, it may validate the importance of this group in forming complementary associations with heme based on interaction study results. Dimer **2** has the shortest distance between its functional units in the series (four atoms). Like dimers **3** and **4**—but unlike dimer **1**—it contains an oxygen that forms an ester group. The importance of this feature for heme interaction will be evaluated. The homologous series (dimers **2**, **3**, and **4**) allowed us to assess how different lengths of the central spacer groups affected the formation of 2:1 complexes. Additionally, two nondimeric derivatives of 3-pyridinopropanol, compounds **9** and **10** (Figure 1C), were designed to evaluate their interactions and affinity with hematin. The structures of these compounds contain structural units found in dimers **1** and **2**, respectively, and terminally present a succinic group with a free carboxyl group.

**Figure 1:**
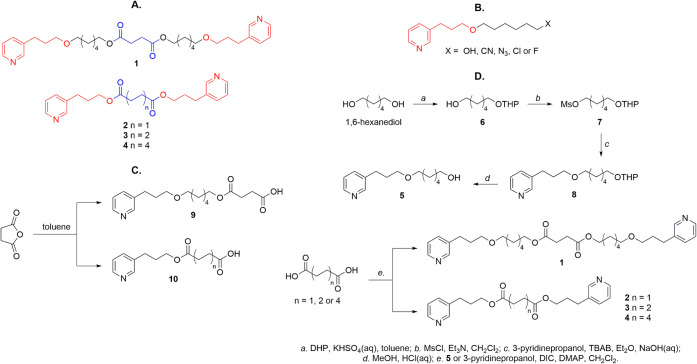
A. Dimeric derivatives of 3-alkylpyridine, B. compounds from the precursor study, C. nondimeric derivatives of 3-pyridinopropanol structures, and D. synthetic route of the compounds.

An initial *in silico* calculation was performed to assess the potential interactions between the proposed molecules and the heme group. The data computed at the B97–3c/def-TZVP level of theory were included in Table 1. It is important to mention that between the **1-heme** and **4-heme** complexes, we have an increase in the chain size separating the two Fe­(protoporphyrin) IX groups (heme groups). The complex identified as **9-heme** presents only one pyridine moiety connected to the heme group instead of two pyridine groups (**1-heme** and **4-heme**). Therefore, two effects were investigated theoretically: (i) the size of the chain connecting two heme groups and (ii) the coordination of two or one pyridine moiety to the heme group. Both trends were also analyzed based on experimental perspective.

**Table 1:** Complexation Energies Evaluated at GFN2-xTB Semiempirical Method and B97-3c Functional for Triple Zeta (Def2-TZVP) Basis Set Quality for the complexes Comprising Two or One Heme Group with Distinct Connecting Chains^a^ ^b^

		B97–3C/def-TZVP	
Complexes	Chain size (Å)^c^	ΔE	ΔG^0^
**1-heme**	36.3	–75.24	–27.20
**4-heme**	25.5	–68.02	–21.50
**9-heme**	20.3	–46.19	+0.70

a Values in kcal mol^–1^.

b Gibbs free energies were evaluated at 298.15 K and 1 atm.

c Distance between the atoms coordinated to the heme group was obtained at B97–3c/def-TZVP optimized geometries. (For **1-heme** and **4-heme**, the distances correspond to the distance between nitrogen atoms of the pyridine moiety.)

According to Table 1, both ΔE_ele‑nuc_ and ΔG^0^ attest that the increase in the chain size between both heme groups attached to two pyridine moiety increases stability. The increase in stability is identified because more negative values of ΔE_ele‑nuc_ and ΔG^0^ (see Table 1) were computed for complexes with higher chain size. As can be seen, the order of the chain size corresponds to **4-heme** < **1-heme**. At the B97–3c/def-TZVP level of theory, the ΔG^0^ values correspond to −21.5 and −27.20 kcal mol^–1^ for **4-heme** and **1-heme**, each one with a chain size corresponding to 25.5 and 36.3 Å, respectively (Figure 2). Therefore, ΔG^0^ becomes more negative when the distance of the atoms connected to the heme group decreases.

**Figure 2:**
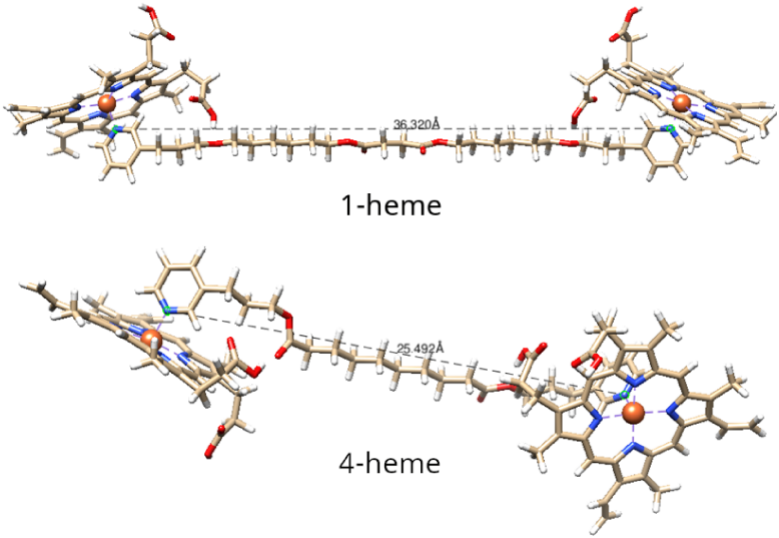
Complex **1-heme** and **4-heme** structures and distances between the atoms coordinated to the heme group were obtained at B97–3c/def-TZVP optimized geometries.

With a focus on the **9-heme** complex, the size of the chain for this complex is 20.3 Å (Figure 3). From Table 1, the **9-heme** system presents the less negative values for ΔEele-nuc and ΔG^0^ among all the complexes studied herein. As can be seen, the ΔG^0^ calculated is positive. In general, it can be concluded that the stability is affected by the coordination of two pyridine groups.

**Figure 3:**
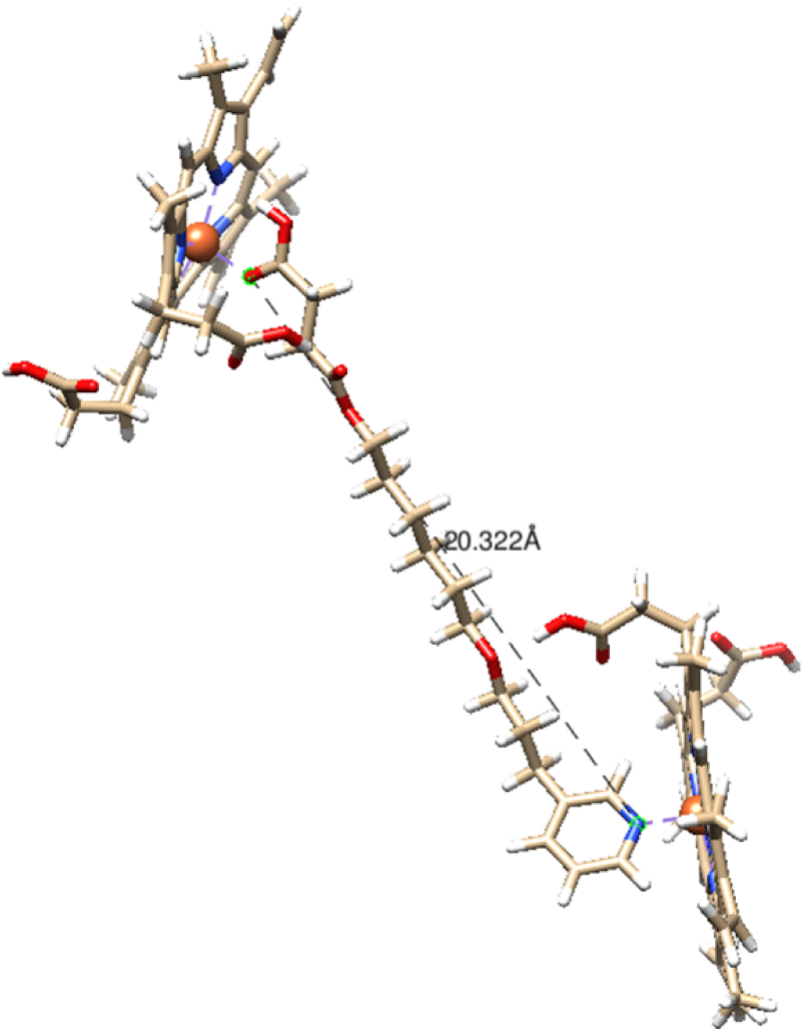
Distance between the nitrogen and oxygen atoms coordinated to the heme group for the **9-heme** complex (chain distance) in Å. Geometry was optimized at B97–3c/Def-2 SVP level of theory.

Finally, according to the theoretical analysis, at the B97–3c level of theory, increasing the chain size between two heme groups increases stability. Furthermore, coordinating the heme groups into two pyridine moieties instead of one increases stability. The theoretical outcomes align well with the experimental information at the most sophisticated level of theory applied.

To assess whether the synthesized compounds are capable of associating with hematin (defined here as the receptor), a model of interaction between these ligands and ferriprotoporphyrin IX was employed, using ultraviolet–visible spectroscopy.^15^ In this type of experiment, small volumes with known ligand concentrations are added while maintaining the receptor concentration constant, and the variation in absorbance in the generated spectra is observed. It is worth noting that interaction studies using hematin are challenging to conduct in pure aqueous solutions due to aggregation and the minimal change in band intensity when it associates with ligands.^18,19^ However, when using an aqueous solution containing 40% DMSO, hematin is maintained in its monomeric form, and its interaction with ligands results in easily observable variations in the Soret band (401 nm). Thus, using this solvent system, both qualitative data, such as the occurrence of ligand–receptor interaction, and quantitative data, such as the calculation of the dissociation constant, can be obtained.^20^

Spectrophotometric titration experiments were performed for dimers **1**-**4** and compounds **5** and **6**. Their respective absorbances versus concentrations for only the molecules showing stronger interactions were plotted in an XY scatter plot, as shown in Figure 4.

**Figure 4:**
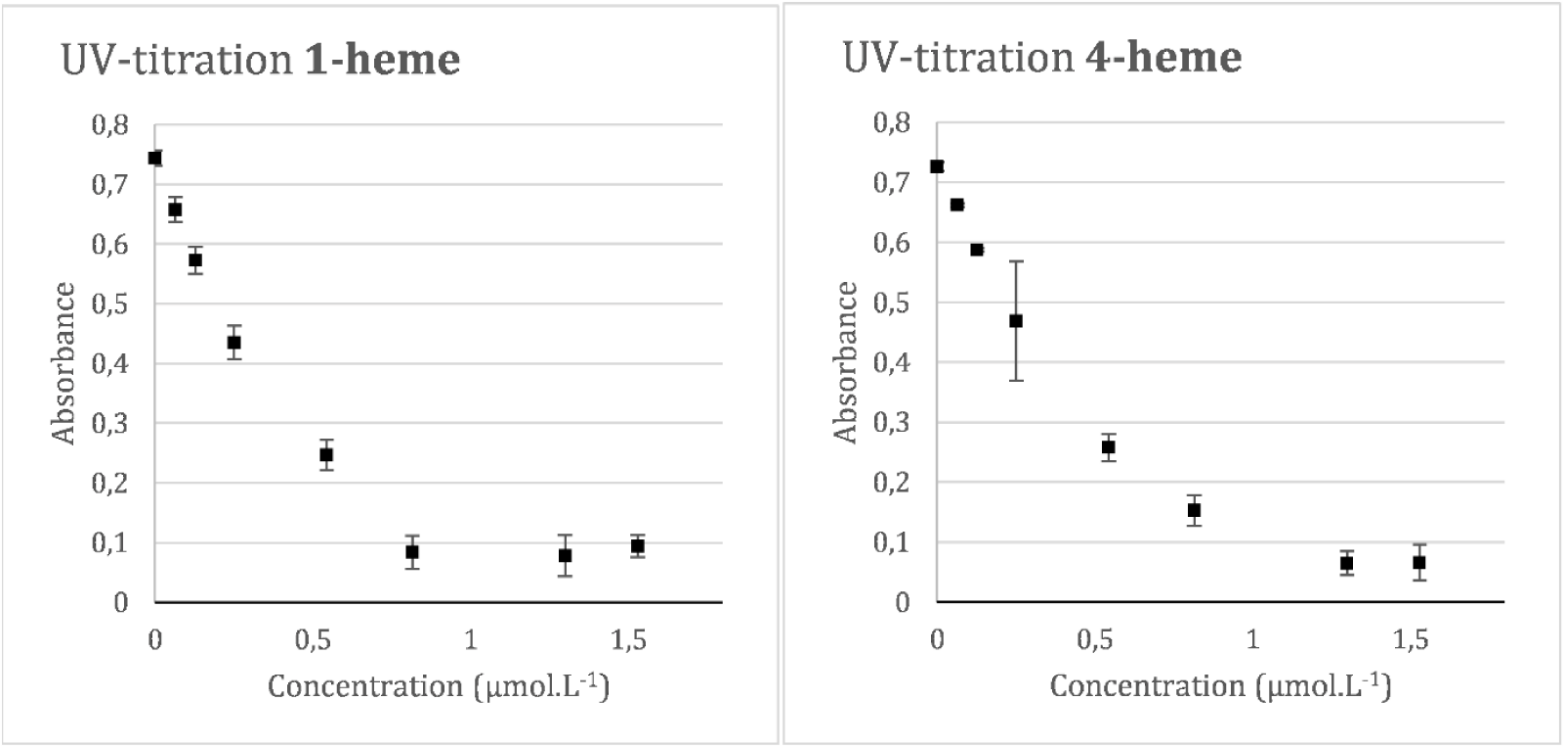
Titration of Hematin (5.0 μM) with increasing concentrations of a) compound **1** (0–1.6 μM) and b) compound **2** (0–1.6 μM) (inset shows a plot of absorbance at 401 nm versus the concentration of compounds).

The first point to highlight is that, for the substances evaluated, the complexes formed between hematin and each of the ligands exhibit a lower molar absorptivity coefficient than that of free hematin. Therefore, when the ligand is added to the receptor solution, two or more species that absorb light at 401 nm are present: free hematin and its ligand-bound forms, which, due to their lower molar absorptivity, contribute to a decrease in absorbance at this wavelength. However, in addition to this, other factors may contribute to the reduction in absorption, such as aggregation induced by the addition of other substances (in this case, the ligands), which could result in precipitation of the species responsible for absorbing radiation at the observed wavelength.^18^

It is important to emphasize that the evaluation of supramolecular interaction between ligands and receptors should consider the shape of the curve in the XY scatter plot. The reversible association between two substances in solution occurs according to an equilibrium between the associated and nonassociated forms. As more ligands are added, the equilibrium shifts, leading to an increased formation of the complex. Since the complex exhibits lower molar absorptivity, with increasing ligand concentration, there is a progressive decrease in the absorbance of the system. However, no matter how much excess ligand is added relative to the receptor, complex formation is limited by the association/dissociation constant and the concentration of the receptor in the medium. In other words, beyond a certain point, even with increased ligand concentration, there is minimal variation in the concentration of the complex formed, and therefore, minimal change in the observed absorbance. Supramolecular association equilibria exhibit nonlinear curves, corresponding to mathematical models such as quadratic and cubic equations. A graph with trends parallel to the *X*-axis, or a plateau, is an important qualitative indicator that a supramolecular association between two substances is occurring.^21–23^

According to Figure 4, only molecules **1** and **4** exhibited sufficiently strong interaction with the heme group to induce the formation of a plateau in their absorbance versus concentration curves. Graphs without a clearly defined region parallel to the *X*-axis also indicate that these ligands are weak, and the curve’s inflection is very subtle due to the low association constant between the two (data not shown).

Quantitative affinity data were calculated only for compounds **1** and **4**, using the Bindfit platform (Thordarson, 2015). The affinities are calculated in terms of the association constant (*K*_*a*_), but in this work, they will be presented as the dissociation constant (*K*_*d*_ = 1/ *K*_*a*_) due to its practical significance. The dissociation constant (*K*_*d*_) represents half the concentration of ligand required to achieve total receptor occupancy. For compounds **1** and **4**, *K*_*d*_ values of 71.17 ± 2.87 and 88.79 ± 3.01 μM were found, respectively.

Considering that both substances are dimeric and symmetrical, and therefore the groups exposed at the ends are identical, a mathematical model for noncooperative 2:1 (heme/ligand) interaction modes was used to calculate the respective *K*_*d*_ values. This model assumes that the binding of the first group does not interfere, either positively or negatively, with the binding of the second.^21^ Therefore, the two dissociation constants, *K*_*d1:1*_ (dissociation constant for the 1:1 complex) and *K*_*d2:1*_ (dissociation constant for the 2:1 complex), have equal values.

This approach proved to be valid since the active chemical groups (pyridinic ring) should not have different affinities toward hematin molecules, and also, considering that for ligands **1** and **4**, which have long spacers, there is minimum distance for that there is no steric hindrance to the formation of the ternary Hematin-Ligand-Hematin complex.

After synthesizing the dimers based on in silico data and in vitro heme group affinity testing, an antimalarial assay was conducted to determine the IC_50_ of the compounds. Surprisingly, IC_50_ values were obtained in the micromolar range, showing modest activity (46.30–140.9 μM), as shown in Table 2. To further refine the study, genotoxicity analysis and in silico permeability assessment were performed.

**Table 2:** Descriptors, In Vitro Data and Average Free Bind Energy Data for Tested Molecules and Reference (chloroquine)^a^

Molecule	MW(g/mol)	*K*_d_(μM)	IC_50_(μM) (*P. falciparum W2*)	IC_50_(μM) (WI-26-VA-4)	SI	ΔG (kJ/mol)	cLogP
**1**	556.73	71.17 ± 2.87	118.28 ± 5.64	>100	0,84	–170.21	5.21
**2**	356.41	NC	79.68 ± 3.55	>100	1,25	–109.14	2.37
**3**	384.46	NC	46.30 ± 3.24	>100	2,16	–107.59	3.26
**4**	440.57	88.79 ± 3.01	82.50 ± 2.72	>100	1,21	–131.09	5.04
**6**	237.25	NC	140.90 ± 5.64	>100	0,71	–88.91	0.99
Chloroquine	319.87	0.3 ± 0.8	0.300 ± 0.016	>100	151,5	–136.59	3.93

a Abbreviations: *K*_d_: Dissociation constant, MW: Molecular weight, IC_50_: Half maximal inhibitory concentration, SI: Selectivity index, ΔG: Free energy change, cLogP: Calculated partition coefficient, NC: not calculated.

Based on the results obtained from the antimalarial activity assays (Table 2), compounds with the lowest IC_50_ values and highest selectivity indices against *P. falciparum* were selected for genotoxicity evaluation. Consequently, compounds **2**, **3**, and **4** were assessed using the *in vitro* alkaline comet assay to determine their potential to induce primary chromosomal damage in V79 cells (Chinese hamster lung cell line).

The results of the genotoxicity evaluation are presented in Figure 5. Compounds **2** and **3** exhibited statistically significant increases in comet scores (*p* < 0.05) only at the highest tested concentrations after 3 h of exposure in V79 cells. In contrast, compound 4 induced comparable effects at all tested concentrations. However, no dose–response relationship was observed under any of the experimental conditions.

**Figure 5:**
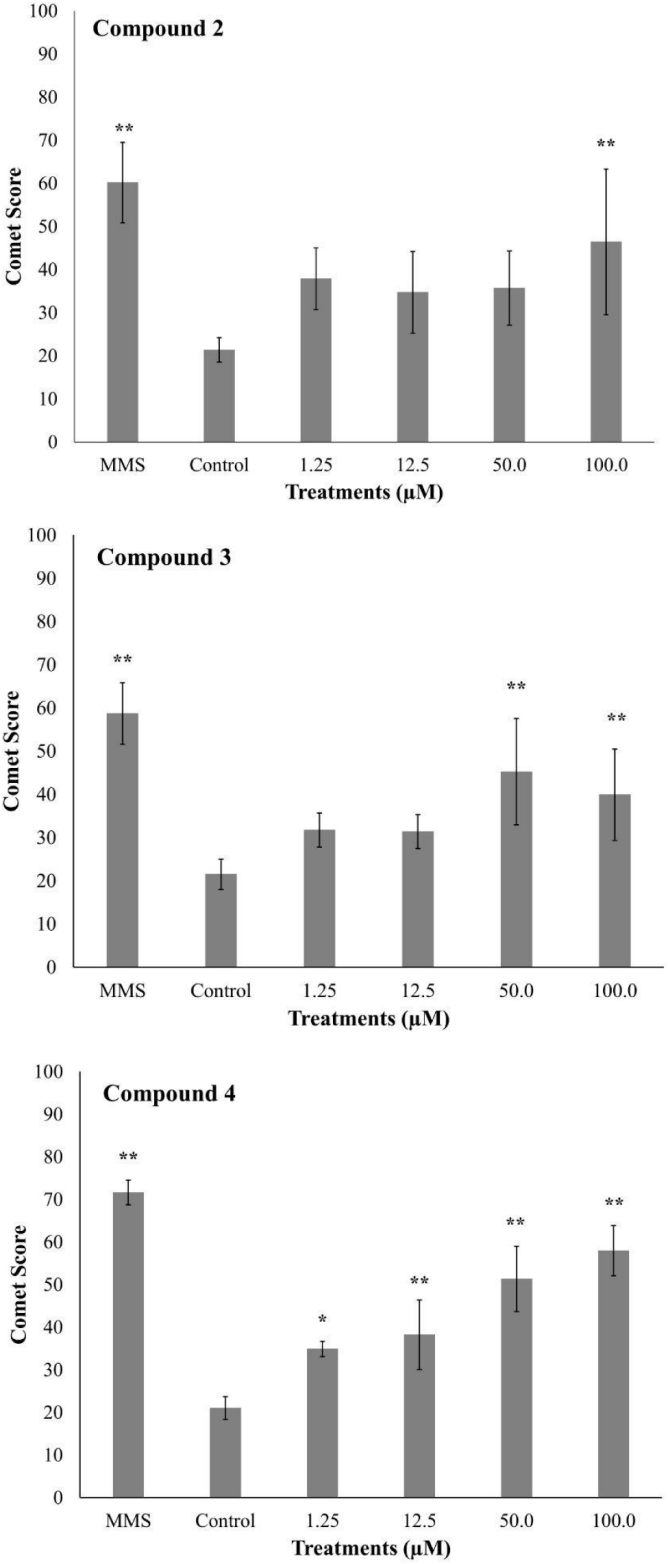
Alkaline Comet Assay. The compounds **2**, **3**, and **4** were evaluated in cell line V79 in treatments of 3 h with different concentrations. MMS: Methylmethanesulfonate 120 μM; Control: serum-free culture media. *: *p* < 0.05; **: *p* < 0.01 (compared with control).

The alkaline comet assay detects agents capable of inducing structural chromosomal alterations, including single-strand breaks, double-strand breaks, and alkali-labile sites.^24^ Although such damage can be repaired by cellular mechanisms, it indicates potential genetic risks associated with exposure. In this context, the results showed that the highest levels of genotoxicity (*p* < 0.01) for compounds **2** and **3** were observed only at concentrations exceeding their respective IC_50_values against *P. falciparum* (Table 2), suggesting a certain safety margin for their use. In contrast, compound 4 exhibited genotoxicity at all tested concentrations, including those below the IC_50_determined for the parasite. This is a noteworthy finding, as the genotoxic potential of the compounds appears to increase with the length of their alkyl chains. Similar results were reported in our previous study^17^ involving synthetic 3-APA derivatives, in which compounds with longer alkyl chains showed greater toxicity against human cell lines.

After evaluating the toxicity profile of the compounds, we sought to investigate whether the low antiplasmodial activity of these compounds could be related to difficulties in crossing phospholipid membranes to reach their molecular target. Various techniques are employed to evaluate or forecast the passive permeability characteristics of molecules across plasma membranes, both in vivo and in vitro. One notable example is the parallel artificial membrane permeability assay (PAMPA), a model of passive, transcellular permeation that evaluates permeability.^25^ A computational approach within Molecular Dynamics (MD) simulations serves to model diffusivity. Additionally, calculating free energy profiles offers a way to establish the best molecular orientations in membranes and compute their permeability coefficients.^26,27^

A bilayer model of one lipid type, 1-palmitoyl-2-oleoyl-*sn*-glycero-3-phosphocholine (POPC – 34:1) was used to evaluate permeability in the bilayer by DM. The passive permeability of drugs can be assessed using simplified models, particularly when considering structures like the blood-brain barrier.^28^

The area per lipid (APL), calculated by considering the x and y axes, was used to assess the stability of the lipid bilayer (Figure 6a) and the partial density profile of the system (Figure 6b). APL) is the cross-sectional area of a system along the bilayer surface plane and can be approximated as the average value of the accessible areas.^29^ The APL value was 64.21 Å^2^. The reported value is in line with that of single lipid type models for POPC, which typically fall between 63 and 66 Å^2^.^30–32^ Moreover, the surface area per lipid (APL) exhibited stability throughout the entire simulation period (Figure 6).

**Figure 6:**
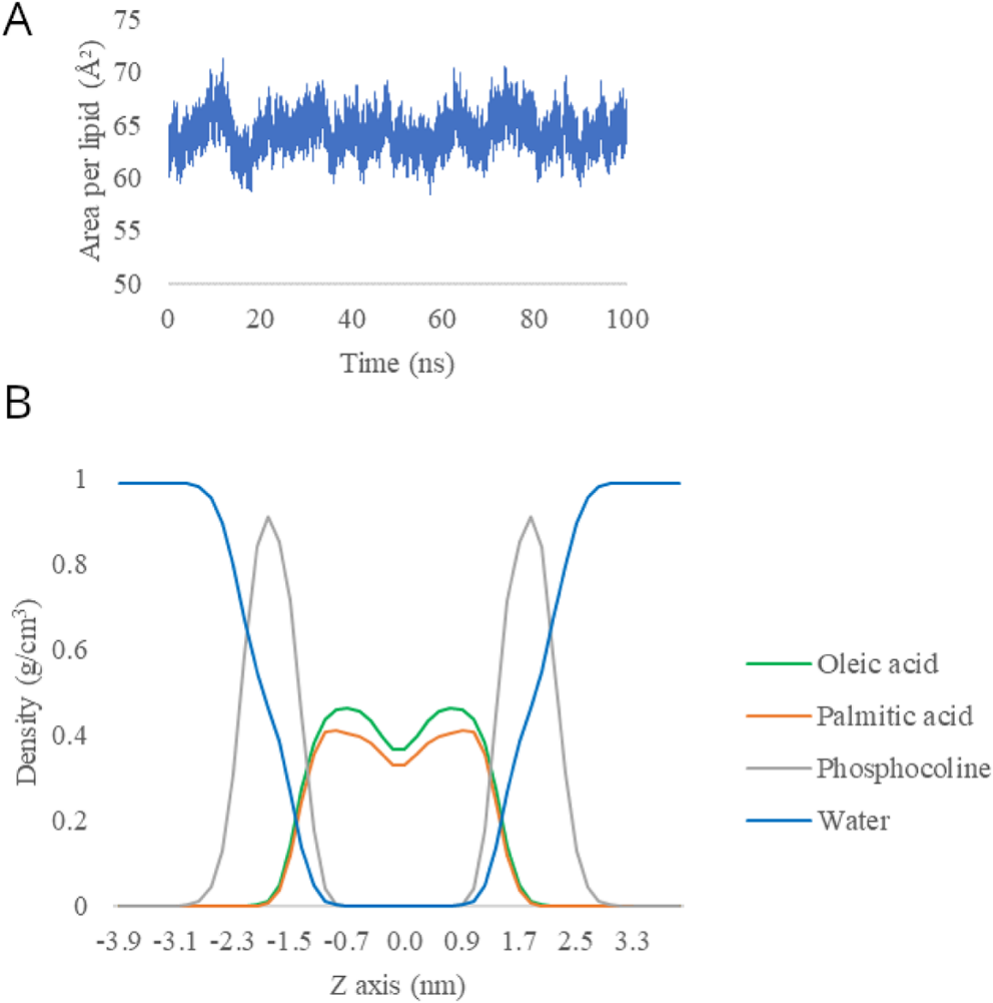
Variation of area per lipid during the trajectory (A) and partial density of the components of the lipid bilayer (B).

The lipid bilayer system presents complexity regarding its composition in different regions and its fluidity. The system’s correct fluidity was ascertained by calculating its partial density profile. The analysis of the phospholipids specifically focused on both the headgroup regions and the hydrocarbon chains. Approximately 90% of the phosphorus atoms belonging to the lipid head groups were found to have a distance from the center of mass that falls between 1.7 and 2.1 nm, a finding consistent with other established models.^33,34^ A reduced density was observed in the bilayer’s central region, which is attributable to the disordered nature of the lipid tails. Throughout the simulation run, the average thickness of the membrane was approximately 3.9 nm. The analysis of density in the interfacial region showed a minimal perpendicular lipid movement and water penetration (Figure 7).

**Figure 7:**
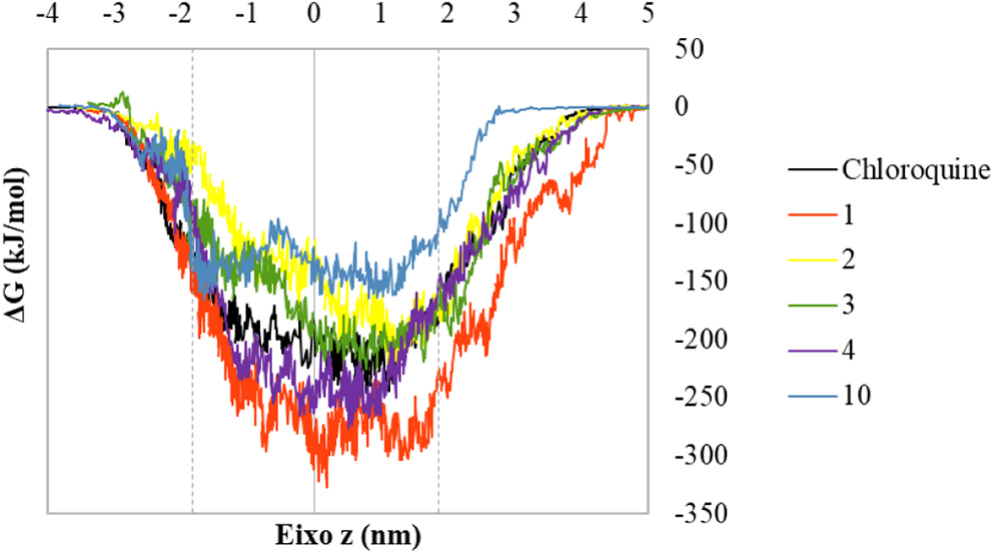
Free energy of molecules **1**, **2**, **3**, **4**, **10,** and chloroquine across the bilayer along the *z* axis.

Food vacuole (FV) of *P. falciparum* is responsible for the hemoglobin degradation process that is a target for several drugs like chloroquine. The permeability profile of chloroquine is widely studied, including by computational models of bilayers containing a single type of POPC lipid.^35^ Therefore, the chloroquine neutral form was used for comparison purposes in permeability analysis.

Simulating a concentration gradient is a computationally expensive process. To overcome this limitation, The ligands were drawn toward the lipid bilayer’s center of mass by a constant artificial potential set at 500 kJ mol^–1^ nm^–1^. Subsequently, the free energy profile was derived using the average force potential (AFP) method, which is founded on constant force pulling. (Table 2 and Figure 7).

Molecule 1 had a lower ΔG value (−170.21 kJ/mol) than choroquine (−136.59 kJ/mol) during the trajectory. Molecule 4 had a similar value to chloroquine (−131.09 kJ/mol). Molecules **2**, **3** and **10** had a higher ΔG value than chloriquine. The evaluation of the ΔG profile during the passage of each molecule across the bilayer allows for the analysis of this behavior (Figure 7).

Due to size, the molecules begin to perform intermolecular interactions with the components of the plasma membrane before the molecules center of mass reach the water/bilayer interface. At this point, there is a decrease in free energy value. A similar behavior was observed just after the molecules cross the lipid bilayer. When crossing the lipid bilayer, all molecules displayed a comparable free energy profile: the free energy value dropped as the molecules approached the water/bilayer interface. The global minimum of the profile was attained upon passing the bilayer’s center of mass (Figure 8). Molecule **1** showed two local minimum when leaving the lipid bilayer which may contribute with a better permeability profile in lipid bilayer.

**Figure 8:**
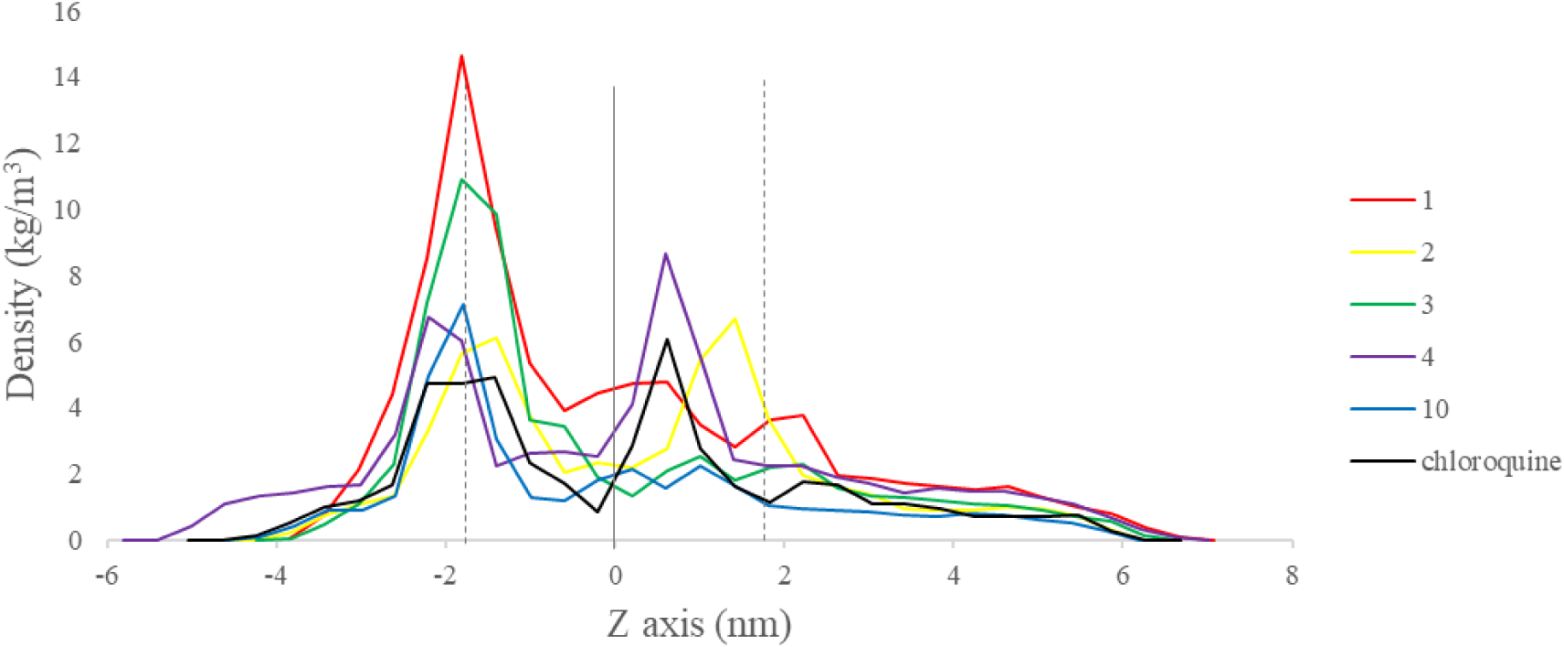
Partial density of molecules **1**, **2**, **3**, **4**, **10,** and chloroquine across the bilayer along the *z* axis.

The passive permeability across lipid bilayer is mainly influenced by lipophilicity. As a quantitative measure of the lipophilicity of bioactive compounds, the clog P value is a widely recognized and used metric. For orally bioavailable drugs, the ideal logP value is between **2** and **5** and have a mass of less than 500 Da.^36^ Molecules **1** and **4** have clogP greater than **5** and the increased lipophilicity may favor the retention of the molecule in the lipophilic phase of the lipid bilayer and hinder permeability (Table 2).

In the constant force method, the speed at which the molecule crosses the plasma membrane can vary. Based on a partial density analysis of the system components along the Z axis (Figure 8), the molecules show a reduction in speed at the water/external lipid bilayer interface. In this sense, molecule **1** is the one that shows the greatest reduction in speed, followed by molecule **3**. This reduction in speed is related to the conformational rearrangements undergone by the molecules when leaving the aqueous phase to enter the lipid bilayer. Molecules **2** and **4** also show a reduction in speed after passing through the center of mass of the lipid bilayer.

When conducting a comprehensive analysis of the data obtained, as shown in Table 1, it is possible to identify an explanation for the antiplasmodial activity of the synthesized substances. Among the molecules prepared compounds **1** and **4** exhibited the strongest interactions with the molecular target, the heme group. However, their cytotoxic activities against Plasmodium ranked among the lowest in the tested series.

On the other hand, considering the simulation of the substances’ passage through the parasite’s lipid bilayer, the ΔG values, which indicate the ease with which these molecules penetrate the bilayer, reveal that compounds **1** and **4** possess the highest penetration capacity. Taking into account that their respective cLogP values are slightly above 5.0, at the threshold of Lipinski’s rule, this suggests they exhibit significant affinity for the lipid phase. This finding implies that these substances have facilitated entry into the lipid bilayers but remain trapped within them. Consequently, they would reach the molecular target at low concentrations, resulting in reduced cytotoxic effectiveness.

This set of data, combined with the evaluation of the number of methylene groups with rotatable bonds (21 and 14, respectively), further highlights unfavorable conditions for the traversal of these substances through the lipid bilayer. This explains their poor performance in cytotoxicity assays against the parasite, despite their demonstrated molecular target interactions in vitro.

## Conclusions

The hypothesized dimeric compounds demonstrated potential for interaction with the target group when evaluated *in silico* using *ab initio* methods. These compounds were subsequently synthesized and tested across various models. In the *in vitro* assay for heme group binding, a favorable interaction with the molecular target was confirmed; however, their antiplasmodial activity was lower than expected. Complementary studies on membrane translocation revealed that the compounds are capable of entering the lipid bilayer but exhibit a high energetic barrier to exit. When analyzed in conjunction with logP values and the number of rotatable bonds, it was observed that compounds with higher values for both parameters tend to show reduced cytotoxic activity, likely due to impaired ability to fully traverse the bilipid membranes. This set of data is critical for understanding the mechanism of antimalarial activity and also provides valuable insights for the rational design of new antimalarial candidates by guiding the optimization of molecular structures based on these key physicochemical parameters.

## Methods

### Synthesis Methodology

#### Materials

Reagents were purchased from Sigma-Aldrich and Fluka, and were used without purification. Pyridine and dimethylformamide were dried over molecular sieves (3 Å) for 48 h. The chromatographic columns used in the purification of compounds were carried out using 40–63 μm silica gel (flash silica, Merck) as the stationary phase.

### Synthesis of Bis 6-(3-(Pyridine-3-yl)­propoxy)­hexyl succinate (1)

Compound 5 (0.160 g; 0.67 mmol) and 20 mL of dichloromethane (dry) were added to a 125 mL reaction flask. The system was conditioned to an inert atmosphere, magnetic stirring and room temperature. Then, succinic acid (0.060 g, 0.506 mmol), DMAP (0.013 g, 0.10 mmol) and DIC (0.19 mL, 1.21 mmol) were added. The progress of the reaction was monitored using TLC plates (eluent: ethyl acetate; developer: phosphomolybdic acid, dragendorff and bromocresol green). After 48 h, the reaction medium was transferred to a separatory funnel and approximately 20 mL of distilled water, 30 mL of a saturated sodium chloride solution and 40 mL of chloroform were added. The aqueous phase was extracted three times with chloroform (approximately 30 mL/wash). The organic phase was then pooled and anhydrous magnesium sulfate (MgSO4) was added to remove residual water. Then, simple filtration was performed and the solvents were removed under reduced pressure. The residue was purified using a chromatographic column (flash silica; 20 mm in diameter; approximately 16 cm in height) using a 99:1 mixture of ethyl acetate/methanol as eluent. Compound 1 was obtained as a colorless oil (yield: 4%).

^1^H NMR (CDCl3, 400 MHz) δH 1,30–1,46 (m, 8H, CH2); 1,50–1,76 (m, 8H, CH2); 1,80–2,00 (m, 4H, CH2); 1,55–1,62 (m, 4H, CH2); 2,71 (t, 4H, J = 8,4 Hz, CH2); 3,30–3,52 (m, 8H, CH2O); 4,09 (t, 4H, J = 6,4 Hz, CH2O); 7,10–7,25 (m, 2H, H5py); 7,55 (d, 2H, J = 7,6 Hz, H4py); 8,47 (sL, 4H, H2py e H6py) ppm.

^13^C NMR (CDCl3, 100 MHz) δC 25,74 (CH2); 25,86 (CH2); 28,52 (CH2); 29,14 (CH2); 29,49 (CH2); 29,60 (CH2); 30,93 (CH2); 64,74 (CH2O); 69,45 (CH2O); 70,84 (CH2O); 123,34 (C5py); 136,11 (C4py); 137,35 (C3py); 147,07 (C6py); 149,74 (C2py); 172,35 (C = O) ppm.

### General Procedure for the Synthesis of Dimers 2, 3, and 4

3-Pyridinepropanol (0.1785 mL; 1.38 mmol) and 20 mL of dry dichloromethane were added to a 50 mL reaction flask. The system was conditioned to an inert atmosphere, magnetic stirring and room temperature. Then, dicarboxylic acid (1.02 mmol, succinic acid being used for the synthesis of dimer 2, adipic acid for dimer 3 and sebacic acid for dimer 4), DMAP (0.025 g; 0.20 mmol) were added.) and DIC (0.382 mL, 2.44 mmol). The progress of the reaction was monitored using TLC plates (eluent: ethyl acetate; developer: phosphomolybdic acid, dragendorff and bromocresol green). After 42–96h, the reaction medium was transferred to a separatory funnel and approximately 20 mL of distilled water, 30 mL of a saturated sodium chloride solution (NaCl) and 40 mL of dichloromethane were added. The aqueous phase was extracted three times with dichloromethane (approximately 30 mL/wash). The organic phase was then pooled and anhydrous magnesium sulfate (MgSO4) was added to remove residual water. Then, simple filtration was carried out and the solvents were vaporized under reduced pressure. The crude residue was purified using a chromatographic column.

### Bis-3-(pyridin-3-yl)­propyl succinate (2)

Conditions for carrying out the chromatographic column: flash silica; 30 mm in diameter; 16 cm in height, using a 95:5 mixture of ethyl acetate/methanol as eluent. Compound 2 was obtained as a colorless oil (yield 62%)

^1^H NMR (CDCl3, 400 MHz): δH 1,93–2,05 (4H, m, CH2); 2,60–2,74 (8H, m, CH2); 4,13 (4H, t, 6,31 Hz, CH2O); 7,19–7,30 (2H, m, H5py); 7,49–7,53 (2H, m, H4py); 8,42–8,50 (4H, m, H2py e H6py) ppm.

^13^C NMR (CDCl3, 100 MHz): δC 28,94 (CH2); 29,22 (CH2); 29,76 (CH2); 63,61 (CH2O); 123,29 (C5py); 135,73 (C4py); 136,32 (C3py); 147,48 (C6py); 149,76 (C2py); 172,15 (C = O) ppm.

### Bis-3-(pyridine-3-yl)­propyl adipate (3)

Conditions for carrying out the chromatographic column: flash silica; 35 mm in diameter; 16 cm high, using ethyl acetate as eluent. Compound 3 was obtained as a colorless oil (yield: 60%)

^1^H NMR (CDCl3, 400 MHz): δH 1,64–1,72 (4H, m, CH2); 1,91–2,06 (4H, m, CH2); 2,28–2,47 (4H, m, CH2); 2,66–2,76 (4H, m, CH2); 4,11 (4H, t, 7,08 Hz, CH2O); 7,17–7,32 (2H, m, H5py); 7,47–7,55 (2H, d largo, 7,98 Hz, H4py); 8,42- 8,50 (4H, m, H2py e H6py) ppm.

^13^C NMR (CDCl3, 100 MHz): δC 29,29 (CH2); 29,31 (CH2); 29,80 (CH2); 33,74 (CH2); 63,28 (CH2O); 123,28 (C5py); 135,69 (C4py); 136,34 (C3py); 147,51­(C6py); 149,79 (C2py); 173,14 (C = O) ppm.

### Bis-3-(pyridin-3-yl)­propyl decanediodate (4)

Conditions for creating the column: flash silica; 25 mm in diameter; 16 cm high, using ethyl acetate/hexane 7:3 as eluent. Compound 4 was obtained as a colorless oil (quantitative yield).

^1^H NMR (CDCl3, 400 MHz): δH 1,08–1,18 (4H, m); 1,19–1,44 (m, 14H); 1,54–1,84 (m, 12H, CH2); 1,92–2,04 (m, 4H, CH2); 2,30 (t, 4H, J = 8,24 Hz CH2); 2,70 (t, 4H, J = 7, 46 Hz, CH2); 4,10 (t, 4H, J = 6,46 Hz, CH2O); 7,20–7, 25 (m, 2H, H5py); 7,49–7,53 (m, 2H, H4py); 8,43–8,49 (m, 4H, H2py e H6py) ppm.

^13^C NMR (CDCl3, 100 MHz): δC 23,47 (CH2); 24,91 (CH2); 29,06 (CH2); 29,40; 29,92 (CH2); 34,24 (CH2); 42,24; 63,20 (CH2O); 123,37 (C5py); 135,84 (C4py); 136,50 (C3py); 147,53 (C6py); 149,83 (C2py); 173,78 (C = O) ppm.

### Synthesis of 4-Oxo-4-(3-(pyridin-3-yl)­propoxy)­butanoic acid (10)

Succinic anhydride (0.150 g; 1.5 mmol) was added to a 125 mL reaction flask. Then, 8 mL of dry toluene was added and the reaction system was conditioned to magnetic stirring, inert atmosphere and heating at reflux. After complete dissolution of the succinic anhydride, 3-pyridinepropanol (0.2 mL; 1.5 mmol) was added and the temperature was maintained at 100 °C. There was no monitoring of the reaction using CCD plates. After 24 h, the flask containing the reaction medium was taken to the rotavapor to remove excess solvent, and was then stored in the refrigerator for 2 days. Then, a precipitate formed. This precipitate was vacuum filtered and subsequently purified by recrystallization with toluene, with compound 10 obtained as a reddish-white solid (60% yield).

^1^H NMR (CDCl3, 400 MHz) δH 1,85–2,00 (m, 2H, CH2); 2,40–2,60 (m, 2H, CH2); 2,60–2,70 (m, 2H, CH2); 4,10 (t, 2H, 6,4 Hz, CH2O); 7,25–7,35 (m, 1H, H5py); 7,64 (d, 1H, 7,6 Hz, H4py); 8,38–8,52 (m, 2H, H2py e H6py); 12,26 (sL, 1H, COOH) ppm.

^13^C NMR (CDCl3, 100 MHz) δC 28,44 (CH2); 28,71 (CH2); 28,75 (CH2); 29,48 (CH2); 63,04 (CH2O); 123,43 (C5py); 135,84 (C4py); 136,61 (C3py); 147,24 (C6py); 149,61 (C2py); 172,09 (C = O); 173,45 (C = O) ppm.

Extensive descriptions of methods and NMR spectra are included as Supporting Information.

## Computational Methods

### Dimers and Heme Interaction

#### Theoretical Calculations

The geometries of the representative complexes, labeled d1, d4, and d9, were optimized without constraints and subjected to vibrational frequency analysis at the semiempirical GFN2-xTB level of theory, employing the xTB code version 6.4.1. The calculations were performed with appropriate charge and spin multiplicity assignments. The optimized geometries were subsequently subjected to unrestricted reoptimization and vibrational frequency analysis employing the B97–3c density functional^3^ and a triple-ζ def2-TZVP basis set, as implemented in the ORCA 5.0 software package.^5^ The gas-phase Gibbs free energy change (ΔG°) for each complex formation was calculated at standard temperature and pressure (298.15 K, 1 atm) using eq 1, which is derived from Statistical Thermodynamics. This calculation considered the electronic and nuclear repulsion energy (ΔE_elec‑nuc_) and thermal corrections (ΔG_T_) derived from the balanced chemical equation.

The theoretical data for analysis, the gas-phase Gibbs free energy (ΔG^0^), was computed assuming the balanced chemical equation for the formation of each complex and contributions of the electronic plus nuclear repulsion energy for the reaction (ΔE_ele‑nuc_) and thermal correction to Gibbs free energy (ΔG_T_) according to, at 298.15 and 1 atm.

1 ΔG0=ΔE(ele−nuc)+ΔGT

### Permeability Evaluation Model in Plasma Membrane

The CHARMM-GUI server^37,38^ was used to build the lipid bilayer model. The phospholipid 1-palmitoyl-2-oleoyl-*sn*-glycero-3-phosphocholine (POPC – 34:1) was incorporated into the system. The second water phase was created with an extended length along the *z*-axis. This configuration was necessary to guarantee that molecules could completely traverse the membrane before reaching 50% of the total length of the simulation box on the *z*-axis, which is a known limitation of the fixed-referential constant-force pull strategy. The overall system was composed of 72 phospholipids, the ligands being studied, and approximately 13,803 TIP3P-like water molecules.

The protocol commenced with 1000 cycles of steep descent energy minimization, followed by NPT (isothermal and isobaric) equilibration cycles. A subsequent production step was then executed to assess the lipid bilayer’s stability by analyzing several parameters: the area per lipid (APL) measured on the x and y axes, the average density of the system components along the *z*-axis, and the membrane thickness variation throughout the trajectory. This molecular dynamics protocol utilized the GROMACS 5.1.2 package, employing parameters set to T = 303 K and pH 7.5. Finally, the free energy over time was evaluated using the g_mmpbsa tool.

### Mean Force Potential Calculation

The topology of the ligands was determined using ACPYPE.^39^ Subsequently, the specialized force fields—namely, the CHARMM-GUI Amber-based membrane and the ACPYPE GAFF-based ligands—were manually combined. This merging step was executed just prior to the ligand’s introduction into the system, which involved substituting an equivalent volume of water molecules. To ensure the absolute coordinate reference for the constant-force pulling, energy minimization and equilibration cycles were performed with positional restraints applied to the ligand under NVT conditions, preceding the productive step. The production dynamics involved an NVT pulling simulation where a potential force of 500 kJmol^–1^nm^1^ was exerted upon the molecule along the *z*-axis, targeting the center of mass of the lipid bilayer.

### Binding Free Energy

The g_mmpbsa tool was used to compute the system’s binding free energy via the molecular mechanics Poisson–Boltzmann surface area MM/PBSA method.^40^ The binding free energy of the bilayer with ligands in solvent can be expressed:

2 ΔGbinding=Gcomplex−(Gbilayer+Gligand)

In this context, Gcomplex represents the total free energy of the bilayer-ligand system, while Gbilayer and Gligand(s) denote the total free energies of the isolated bilayer and the ligands in the solvent, respectively. Conventionally, the binding free energy ΔGbinding defined in (eq 3) encompasses the entropic (TΔS) and enthalpic (ΔH) contributions. The enthalpic term, detailed in (eq 4), consists of the potential energy derived from molecular mechanics in a vacuum (GMM) and the solvation free energy (Gsolvation). GMM (eq 5) is further partitioned into the noncovalent interaction energies, specifically van der Waals (EvdW) forces and electronic interactions (Eelec). Meanwhile, Gsolvation (eq 6) is comprised of polar Gpolar and nonpolar Gnonpolar components. The Gpolar term was determined by solving the Poisson–Boltzmann equation, whereas Gnonpolar was calculated via (eq 7). In this equation, SASA stands for the solvent accessible surface area, and the terms Gsolvation and the coefficient are empirical constants utilizing their default values. Consequently, the binding free energy for each individual Gcomplex, Gbilayer, and Gligand was estimated by applying (eqs 3–7).

3 ΔGbinding=ΔH−TΔS

4 ΔH=(GMM)+(Gsolvation)

5 GMM=EvdW+Eelec

6 Gsolvation=Gpolar+Gnonpolar

7 Gnonpolar=γSASA+β

Accordingly, ten MD snapshots were extracted every 1 ns from the production trajectories (spanning 7–50 ns) for the binding free energy calculations. The electrostatic (Eelec) and van der Waals (EvdW) contributions were obtained using the Coulomb and Lennard–Jones (LJ) potential functions, respectively. The polar solvation energy (Gpolar) was computed within a grid box defined by an expansion factor of 2 (cfac = 2) and an additional 20 Å to the molecular dimensions (fadd = 20), using a 0.150 M NaCl solvent (radiiNa = 0.95 Å; radiiCl = 1.81 Å) and a dielectric constant of 80.0, according to the Debye–Hückel approximation. The nonpolar solvation energy (Gnonpolar) was then estimated using a solvent-accessible surface area (SASA) model, applying the default solvent surface tension parameters (γ = 0.02267 kJ·mol^–1^·Å^– 2^; β = 3.84928 kJ·mol^– 1^).^41–43^

### UV–Vis Spectroscopic Titrations

#### Preparation of Solutions

##### DMSO/HEPES Buffer Solution (40%)

1.1906 g of HEPES was dissolved in 5 mL of water. This HEPES solution was transferred to a 250 mL volumetric flask containing 100 mL of dimethyl sulfoxide (DMSO). The volume was brought up to 250 mL, and the solution was homogenized. The pH of the HEPES buffer was adjusted to 7.4. The solution was stored in the refrigerator, protected from light.

##### Hematin Stock Solution (2.6 mmol L^‑1^) (SEhm)

17.2 mg of hemin was weighed and quantitatively transferred to a 10 mL volumetric flask. The flask was then filled to the meniscus with HEPES buffer solution (pH 7.4). The solution was stored in the refrigerator, protected from light.

##### Hematin Analysis Solution (5 μmol L^‑1^) (SAhm)

The hematin analysis solution was prepared by transferring 46 μL of the hematin stock solution (SEhm), using a micropipette, to a 25 mL volumetric flask. This flask was then filled to the meniscus with HEPES buffer solution (pH = 7.4). This solution was prepared on the day of analysis.

##### Stock Solutions of Ligands (SE)

The ligand stock solutions were prepared by dissolving masses of each compound ranging from 11.0 mg to 30.1 mg in 800 μL of DMSO.

##### Ligand Analysis Solutions (SA)—General Procedure

The analysis solutions were prepared by diluting the ligand stock solutions in hematin analysis solution (SAhm), so that the ligands had a concentration of 8.10 mmol L-1. All solutions were prepared on the day of analysis.

##### UV–Vis Spectroscopic Titrations

Two cuvettes were prepared for the UV–vis spectrophotometry analysis: one to serve as the blank solution and the other for the actual analysis. Approximately 2.5 mL of the HEPES buffer solution was placed in the blank cuvette, while 2.5 mL of the hematin analysis solution (SAhm) was placed in the analysis cuvette. A thermal control was applied to maintain both cuvettes at 25 °C, and the analysis cuvette was subjected to magnetic stirring. Sequential additions of the ligand analysis solutions were made in the analysis cuvette, following the order: + 20 μL, + 20 μL, + 40 μL, + 100 μL, + 100 μL, + 200 μL, + 100 μL. It is important to note that after each addition, a 1 min waiting period was observed before starting the scan, which was performed between wavelengths of 200 and 500 nm. Absorbance at 401.40 nm was measured in each reading. The experiments related to dimers 1 and 4 were performed in triplicate.

##### Determination of Dissociation Constants (*K*_d_) for Compounds 1, 4, and Chloroquine

The dissociation constants were calculated using nonlinear regression through the online software available on the BindFit platform (http://app.supramolecular.org/bindfit/). The calculation parameters for dimers 1 and 4 were: Filter: UV 2:1, K11 indication = 1000 mol-1, noncooperative interaction mode, Nelder–Mead method, no dilution factor correction, and no subtraction of initial values. For chloroquine, the parameters were: Filter: UV 1:1, K11 indication = 1000 mol-1, Nelder–Mead method, no dilution factor correction, and no subtraction of initial values.

## Biological Assays

### In Vitro Activity of the Compounds

Chloroquine-resistant (W2) strains of P. falciparum were grown in human red blood cells under in vitro conditions described by Trager and Jensen (1976). The parasites were maintained in Petri dishes at a hematocrit of 5%, using a complete RPMI 1640 medium supplemented with 25 mM Hepes, 21 mM sodium bicarbonate, 300 μM hypoxanthine, 11 mM glucose, 40 μg/mL gentamicin, and 10% (v/v) heat-inactivated human plasma. Cultures synchronized with sorbitol treatment, presenting 0.5% ring-stage parasitemia and 2% hematocrit, were distributed in 96-well plates at 180 μL per well. The tested compounds were added in triplicate (20 μL per well) at serial concentrations ranging from 50 to 0.1 μmol/L. Control wells contained infected erythrocytes without any compound (negative control), while chloroquine was included as the positive control in all experiments. Additionally, 180 μL of noninfected erythrocytes were added to six wells to correct for autofluorescence.

The plates were incubated at 37 °C for 48 h. Following incubation, the supernatant was removed and 150 μL of 1X phosphate-buffered saline (PBS) was added to each well. The plates were then centrifuged at 700 g for 5 min, the supernatant was again discarded, and 120 μL of a lysis buffer containing SYBR Safe (20 mM TRIS-base, 5 mM EDTA, 0.008% (w/v) saponin, 0.08% (v/v) Triton X-100, and 0.2 μL/mL SYBR Safe) were added. After erythrocyte lysis, the wells were homogenized, and 100 μL from each well were transferred to a new plate containing 100 μL of PBS. Fluorescence was measured after 30 min of incubation in the dark using a fluorimeter set to 484 nm excitation and 535 nm emission. The results were expressed as the concentration of compound that reduced parasite viability by 50% (IC50), determined through nonlinear regression analysis of the dose–response curve.

### In Vitro Cytotoxicity

The in vitro cytotoxicity of each compound was evaluated using WI-26VA4 human pulmonary fibroblast cells (ATCC CCL-95.1, USA) through the MTT assay. Cells were cultured in RPMI-1640 medium (Sigma-Aldrich, St. Louis, Missouri, USA) supplemented with 10% heat-inactivated fetal bovine serum and seeded into 96-well plates. The tested compounds were prepared in serial dilutions ranging from 200 to 0.2 μmol/L and incubated with the cells for 24 h at 37 °C in a humidified atmosphere containing 5% CO_2_. Optical density readings were obtained at 540 nm to measure signal and background using a microplate reader (Spectra Max340PC384, Molecular Devices, Sunnyvale, California, USA). The concentration causing 50% cell death (IC50) was determined according to previously described procedures.

### Selectivity Index

The selectivity index (SI) for each tested compound was determined as the ratio between its cytotoxic and antiplasmodial activities. Compounds with SI values greater than 10 were regarded as noncytotoxic, while those with values below 10 were considered toxic. The SI was calculated according to the following equation:

SI=IC50cells/IC50P.falciparum

### In Vitro Genotoxicity—Alkaline Comet Assay

The comet assay used to assess DNA damage was carried out following the procedure described by Singh et al. (1988), with minor modifications. V79 cells (Chinese hamster lung fibroblasts) were cultured in 25 cm^2^ flasks containing 5 mL of DMEM/F12 (1:1) medium supplemented with 10% fetal bovine serum (FBS), 1.2 g/L sodium bicarbonate, and an antibiotic-antimycotic solution, and maintained at 37 °C in an atmosphere of 5% CO_2_ and 95% humidity.

For the experiments, 4 × 10^5^ cells per well were seeded in 24-well plates containing 500 μL of culture medium and incubated for about 24 h. After this incubation period, the cells were exposed for 3 h to compounds 2, 3, and 4 at concentrations of 1.25, 12.5, 50, and 100 μM in culture medium without FBS. The negative control group received FBS-free culture medium, while the positive control was treated with 120 μM methylmethanesulfonate (MMS).

Following the treatment period, the cell cultures were trypsinized, centrifuged, and resuspended in culture medium. Cell suspension aliquots were mixed with low-melting-point agarose (0.5%). Each aliquot was layered onto microscope slides precoated with a base layer of normal-melting-point agarose (1.5%), covered with coverslips, and refrigerated for 30 min to allow agarose solidification. Coverslips were then removed, and the slides were transferred to a freshly prepared, chilled lysis solution (2.5 M NaCl, 100 mM EDTA, 10 mM Tris, pH 10, with 1% Triton X-100 and 10% DMSO).

After 1 h in the lysis solution, the slides were placed in a horizontal electrophoresis chamber filled with a freshly prepared, chilled alkaline buffer (1 mM EDTA and 300 mM NaOH, pH > 13) for 20 min. Electrophoresis was conducted at 1 V/cm and 300 mA for an additional 20 min. Following electrophoresis, slides were neutralized in 0.4 M Tris buffer (pH 7.5), fixed with absolute ethanol, and air-dried at room temperature.

For microscopic analysis of nucleoids, slides were stained with ethidium bromide solution (20 μg/mL), covered with coverslips, and observed under a fluorescence microscope (Axioscope – Zeiss) equipped with an excitation filter of 515–560 ηm and a barrier filter of 590 ηm at 400× magnification. Two slides per treatment were analyzed, with 100 nucleoids counted per slide and categorized into comet classes from 0 to 4, where class 0 indicates no damage and class 4 represents maximal damage.^44^

Using these values, the comet score for each treatment was calculated according to the equation by Møller et al. (2023):

Score=(0×A)+(0.25×B)+(0.50×C)+(0.75×D)+(1×E)

where A, B, C, D, and E represent the number of nucleoids in each class (0 to 4, respectively).

All experiments were independently repeated three times, and during each repetition, cell viability following treatment was assessed using trypan blue staining.

Comet assay data were processed using GraphPad Prism version 7.0 and Microsoft Excel (Microsoft 365). Statistical differences were determined by one-way analysis of variance (ANOVA), followed by Tukey’s post hoc test for multiple comparisons. Dose–response relationships were examined using a linear regression model, and the Pearson correlation coefficient (r) was used to evaluate the strength and direction of the correlation. For regression analysis, an R^2^ value above 0.8 and a steeper regression slope were considered indicative of a stronger fit to the linear predictive model. Results are expressed as mean ± standard deviation (SD), with statistical significance set at *p* < 0.05.

## Supplementary Material



## Notes

Synthetic procedures, materials, and methods for the prepared compounds, including their characterization by ^1^H and ^13^C NMR spectroscopy (PDF)
