# Supplementary material for: Antimalarial Potential of Heme-Targeting Dimeric Compounds: Binding Efficacy vs. Membrane Retention Effects
Source: ACS Omega. 2026 Jan 8;11(3):3889–901. doi: 10.1021/acsomega.5c06934 (PMC12854372; doi:10.1021/acsomega.5c06934)
Supplement: Supplementary file 1 [file ao5c06934_si_001.pdf]

Antimalarial Potential of Heme-Targeting Dimeric Compounds: Binding Efficacy vs. Membrane Retention Effects

Victor Matheus Kemmer[a], Fabricio Santos[a], Fernanda Alice de Oliveira[b,c], Ana Claudia de Sousa Pinto[b,c], Amanda Luisa da Fonseca[b,c], Letícia Aparecida da Silva[b,d], Helen Gonçalves Marques[b,e], Fabio Vieira dos Santos[b,e], Cleber Paulo Andrada Anconi[f], Franco Henrique Andrade Leite[g], David Bacelar Costa Junior[g], Fernando de Pilla Varotti[b,c], Clébio Soares Nascimento Júnior[b,d], Luciana Guimarães[b,d], Gustavo Henrique Ribeiro Viana[b], Renato Márcio Ribeiro-Viana[b,h], Anna Paola Butera[a,b*]

[a] *Departamento de Química, Universidade Estadual de Londrina, Londrina, 86057-970, Brazil*

[b] *Núcleo de Pesquisa em Química Biológica (NQBio), Universidade Federal de São João del Rei, Divinópolis, 35501-296, Brazil*

[c] *Laboratório de Bioquímica Medicinal, Universidade Federal de São João del Rei, Divinópolis, 35501-296, Brazil*

[d] *Departamento de Ciências Naturais, Universidade Federal de São João del-Rei, Campus Dom Bosco, São João del Rei, 36301-160, Brazil*

[e] *Laboratório de Biologia Celular e Mutagênese, Universidade Federal de São João del Rei, Divinópolis, 35501-296, Brazil*

[f] *Departamento de Química, Instituto de Ciências Naturais, Universidade Federal de Lavras, Lavras, 37200-900, Brazil*

[g] *Laboratório de Químioinformática e Avaliação Biológica, Universidade Estadual de Feira de Santana, Feira de Santana, 44036-900, Brazil*

[h] *Programa de Pós-graduação em Ciência e Engenharia de Materiais, Universidade Tecnológica Federal do Paraná, Londrina, 86036-370, Brazil*

*Email: paolabut@uel.br

Analytical methods
NMR analysis

1H and ^{13}C spectra were recorded on a Bruker Avance III 400 MHz spectrometer operated at a frequency of 400.6 MHz for 1H, and at 100 MHz for 13C using a 5 mm inverse probe, BBI, at 25°C. Chemical shifts (d) for 1H spectra are expressed in ppm and calibrated according to the residual solvent signal (CHCl3; d = 7.26 ppm; HOD: d = 4,76 ppm). Chemical shifts (d) for 13C spectrum are expressed in ppm and calibrated according to the solvent signal (CDCl3; d = 77.00 ppm).

Synthesis

Synthesis of 6-((tetrahydro-2H-pyran-2-yl)oxy)hexan-1-ol

1,6-Hexanediol (0.120 g; 0.59 mmol) was added to a 25 mL reaction flask. Then, 34.2 mL of toluene and 2.4 mL of a solution of potassium hydrogen sulfate (KHSO4) at 2.5 mol.L-1 were added. Then, 0.3 mL of DHP (3.29 mmol) was slowly added. The reaction system was conditioned to magnetic stirring and room temperature. The progress of the reaction was monitored using TLC plates (eluent: ethyl acetate/hexane 1:1; developer: phosphomolybdic acid). After 2 h of reaction, without total consumption of the starting material having been observed, extraction was carried out with distilled water and dichloromethane until the medium was neutralized (three washes, approximately 30 mL of water/wash). Anhydrous magnesium sulfate (MgSO4) was added to the organic phase, which was then filtered and the solvent was removed by distillation under reduced pressure. The residue was purified by column chromatography (flash silica; 20 mm in diameter and approximately 16 cm in height), using a 7:3 hexane/ethyl acetate mixture as eluent. Compound 6 was obtained as a colorless oil (72% yield).

^{1}H NMR (CDCl3, 400 MHz) δH 1,32-1,46 (m, 4H, CH2); 1,47-1,65 (m, 8H, CH2 e CH2-THP); 1,66-1,76 (m, 1H, CH2); 1,78-1,90 (m, 1H, CH2); 3,39 (dt, 1H, J=9,6 Hz, 6,6 Hz, CH2O-THP); 3,46-3,55 (m, 1H, CH2O); 3,64 (t, 2H, J=6,6 Hz, CH2OH); 3,74 (dt, 1H, J=9,6 Hz, 6,8 Hz, CH2O-THP); 3,81-3,92 (m, 1H, CH2O); 4,55-4,59 (m, 1H, CH-THP).

^{13}C NMR (CDCl3, 100 MHz) δC 19,66 (CH2-THP); 25,44 (CH2); 25,50 (CH2-THP); 25,98 (CH2); 29,63 (CH2); 30,73 (CH2-THP); 32,64 (CH2); 62,35 (CH2O); 62,80 (CH2OH); 67,48 (CH2O-THP); 98,86 (CH-THP).

Synthesis of 6-((tetrahydro-2H-pyran-2-yl)oxy)hexyl methanesulfonate

Compound 6 (0.64 g, 3.16 mmol) was added to a 125 mL flask. Then, 10 mL of dry dichloromethane was added and the reaction medium was conditioned with magnetic stirring. Then, in an ice bath, 1.8 mL of triethylamine (12.93 mmol) and, dropwise, 0.5 mL of mesyl chloride (6.49 mmol) were slowly added. After the additions were made, the reaction medium was slowly returned to room temperature. The progress of the reaction was monitored using TLC plates (eluent: ethyl acetate/hexane 1:1; developer: phosphomolybdic acid). After 18 h, total consumption of the starting material was observed, then extraction was carried out with ice-cold distilled water and dichloromethane (three washes, approximately 30 mL of dichloromethane/wash). Anhydrous magnesium sulfate was added to the organic phase, which was then filtered and distilled under reduced pressure. The residue obtained was purified by column chromatography (flash silica; 20 mm in diameter; approximately 15 cm in height), using

a 7:3 hexane/ethyl acetate mixture as eluent. Compound 7 was obtained as a yellowish oil (75% yield).

^{1}H NMR (CDCl3, 400 MHz) δH 1,36-1,48 (m, 4H, CH2-THP); 1,49-1,66 (m, 6H, CH2 e CH2-THP); 1,73-1,82 (m, 2H, CH2); 3,05 (s, 3H, CH3); 3,39 (dt, 1H, J=9,6 Hz, 6,5 Hz, CH2O-THP); 3,45-3,55 (m, 1H, CH2O); 3,74 (dt, 1H, J=9,6 Hz, 6,7 Hz, CH2O-THP); 3,80-3,93 (m, 1H, CH2O); 4,23 (t, 2H, J=6,6 Hz, CH2OMs) 4,52- 4,60 (m, 1H, CH-THP).

^{13}C NMR (CDCl3, 100 MHz) δC 19,68 (CH2-THP); 25,25 (CH2-THP); 25,42 (CH2); 25,70 (CH2); 29,03 (CH2); 29,48 (CH2); 30,73 (CH2-THP); 37,32 (CH3); 62,40 (CH2O); 67,30 (CH2O-THP); 69,99 (CH2OMs); 98,91 (CH-THP).

Synthesis of 3-(3-((6-((tetrahydro-2H-pyran-2yl)oxy)hexyl)oxy)propyl)pyridine (8)

3-Pyridinepropanol (0.747 mL; 5.79 mmol) was added to a 125 mL reaction flask. Then, diethyl ether (22 mL), tetrabutylammonium bromide (1.092 g; 3.4 mmol), and 50% (m/v) sodium hydroxide solution (11.6 mL) were added. The medium remained under magnetic stirring, semi-open, for 15 minutes. After this period, compound 7 (1.738 g; 6.20 mmol) was added. The system was conditioned at room temperature and magnetic stirring. The progress of the reaction was monitored using TLC plates (eluent: ethyl acetate/hexane 3:7; developer: phosphomolybdic acid). After 70 h, the aqueous phase was extracted three times with ethyl ether (approximately 20 mL/wash). The organic phase was pooled and anhydrous magnesium sulfate (MgSO4) was added to remove residual water. Then, the medium was filtered and the solvent was vaporized using reduced pressure (rotating steam followed by high vacuum). The residue was purified on a chromatographic column (flash silica; 20 mm in diameter; approximately 17 cm in height) using a 7:3 hexane/ethyl acetate mixture as eluent. Compound 8 was obtained as a yellowish oil (38% yield).

^{1}H NMR (CDCl3, 400 MHz) δH 0,75-1,00 (m, 1H); 1,10-1,30 (m, 2H); 1,35-1,45(m, 4H, CH2); 1,45-1,70 (m, 14H, CH2); 1,75-1,90 (m, 4H, CH2); 2,65-2,75 (m,2H, CH2); 3,30-3,56 (m, 8H, CH2O); 3,60-3,70 (m, 1H, CH2O); 3,70-3,80 (m, 1H,CH2O-THP); 3,80-3,92 (m, 1H, CH2O-THP); 4,57 (m, 1H, CH-THP); 7,20-7,30(m, 1H, H5py); 7,50-7,55 (m, 1H, H4py); 8,40-8,48 (m, 2H, H2py e H6py).

^{13}C NMR (CDCl3, 100 MHz) δC 19,68 (CH2-THP); 25,43 (CH2-THP); 25,51;25,98 (CH2); 26,05 (CH2-THP); 26,10; 29,46 (CH2); 29,66 (CH2); 30,72 (CH2-THP); 30,95 (CH2); 32,65; 62,36; 62,86 (CH2O); 67,54 (CH2O-THP); 69,40(CH2O); 70,95 (CH2O); 98,83; 98,87 (CH-THP); 123,28 (C3py); 135,96 (C4py);137,24 (C3py); 147,20 (C6py); 149,88 (C2py)

Synthesis of 6-(3-(pyridin-3-yl)propoxy)hexan-1-ol (5)

Compound 8 (0.804 g; 2.5 mmol) was added to a 125 mL reaction flask. Then, 50 mL of methanol (CH3OH) was added and the system was conditioned to magnetic stirring at room temperature. Finally, 5 mL of a hydrochloric acid (HCl) solution with a concentration of 1.0 mol.L1 was added. The progress of the reaction was monitored using TLC plates (eluent: ethyl acetate; developer: phosphomolybdic acid). After 24 h, the solvent was removed by distillation under reduced pressure and then equal amounts of ethyl acetate and distilled water (30 ml) were added. Then, the medium was alkalinized (20 mL of a 2.0 mol.L-1 NaOH solution was added) and the aqueous phase was extracted with ethyl acetate. The organic phase was combined and anhydrous magnesium sulfate (MgSO4) was added. Once this was done, simple filtration was carried out and the solvent was removed by distillation under reduced pressure.

The crude residue was purified using a chromatographic column (flash silica; 20 mm in diameter; approximately 15 cm in height) using ethyl acetate as eluent. Compound 5 was obtained as a yellowish oil (54% yield).

^{1}H NMR (CDCl3, 400 MHz): δH 1,32-1,47 (m, 4H, CH2); 1,50-1,67 (m, 4H,CH2); 1,89 (m, 2H, CH2); 2,71 (t, 2H, J=7,7 Hz, CH2); 3,35-3,46 (m, 4H, CH2O);3,65 (t, 2H, J=6,5 Hz, CH2OH); 7,16-7,31 (m, 1H, H5py); 7,52 (d, 1H, J=7,7Hz, H4py); 8,39-8,48 (m, 2H, H2py e H6py).

^{13}C NMR (CDCl3, 100 MHz): δC 25,50 (CH2); 25,92 (CH2); 29,37 (CH2); 29,58 (CH2); 30,81 (CH2); 32,66 (CH2); 62,59 (CH2OH); 69,25 (CH2O); 70,78 (CH2O); 123,29 (C5py); 135,99 (C4py); 137,23 (C3py); 147,05 (C6py); 149,78 (C2py).

Synthesis of 4-oxo-4-(6-(3-(pyridin-3-yl)propoxy)hexyl)oxy)butanoic acid (9)

Compound 5 (0.072 g; 0.3034 mmol), succinic anhydride (0.123 g; 1.23 mmol), DMAP (0.045 g; 0.37 mmol) and 10 mL of dichloromethane (dry) were added to a reaction flask. of 50 mL. Then, the reaction system was conditioned to magnetic stirring, inert atmosphere and room temperature. The progress of the reaction was monitored using CCD plates (eluent: ethyl acetate/hexane 8:2, developer: phosphomolybdic acid and bromocresol green). After 48 hours of reaction, when total consumption of the starting material was observed, the solvent was removed from the reaction medium by distillation under reduced pressure. The crude product was taken to a filter column (flash silica; 20 mm diameter; 7 cm height) and three fractions were collected: the first was collected using approximately 40 mL of ethyl acetate/hexane 8:2; the second was collected with approximately 45 mL of ethyl acetate; and the third was collected with methanol. The product of interest was identified in the 2nd fraction, with quantitative yield.

^{1}H NMR (CDCl3, 400 MHz) δH 0,85-0,95 (m, 1H); 1,20-1,29 (m, 2H); 1,30-1,45 (m, 5H, CH2); 1,50-1,59 (m, 5H, CH2); 1,59-1,69 (m, 2H, CH2); 1,84-1,94 (m, 2H, CH2); 2,59-2,72 (m, 4H, CH2); 2,77 (t, 2H, J=5,80 Hz, CH2); 3,20-3,39 (m, 4H, CH2O); 4,15 (t, 2H, J=6,09 Hz, CH2O); 7,29-7,33 (m, 1H, H5py); 7,62 (d, 1H, J=7,64 Hz, H4py); 8,41-8,52 (m, 2H, H2py e H6py) ppm.

^{13}C NMR (CDCl3, 100 MHz) δC 25,40 (CH2); 25,44 (CH2); 28,45 (CH2); 29,15 (CH2); 29,33 (CH2); 29,45 (CH2); 29,67 (CH2); 30,36 (CH2); 64,28 (CH2O); 68,26 (CH2O); 70,65 (CH2O); 123,86 (C3py); 137,49 (C4py); 138,03 (C3py); 145,23 (C6py); 148,47 (C2py); 172,66 (C=O); 175,15 (C=O) ppm.

NMR Spectra

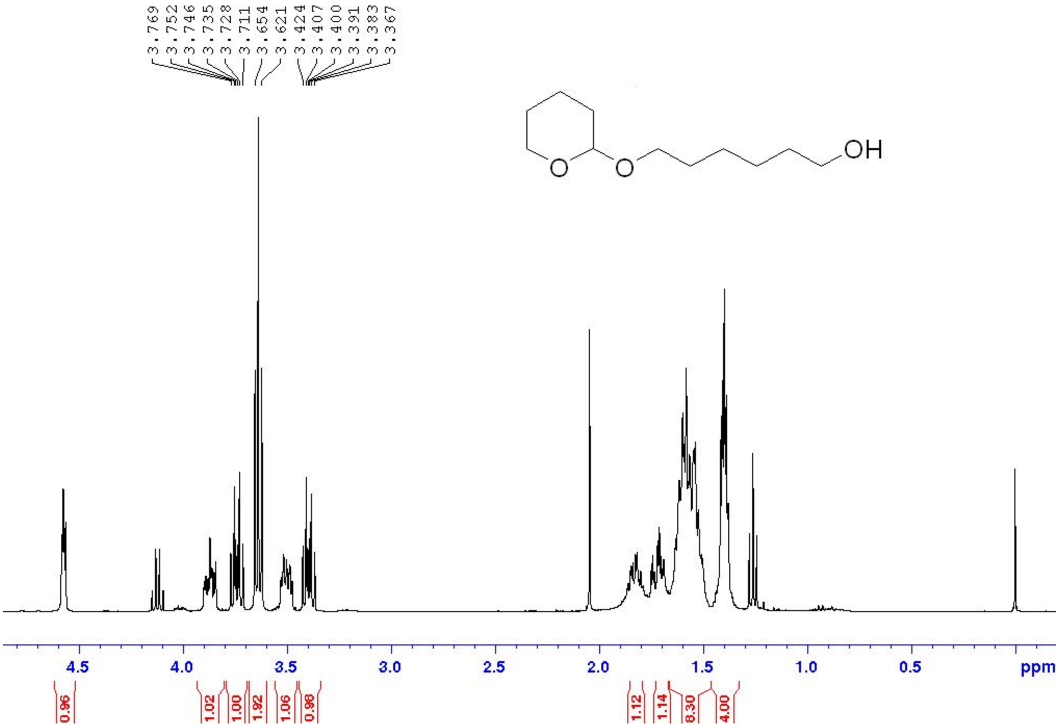

Figure S1- ^{1}H NMR spectrum ($CDCl_3$, 400 MHz)

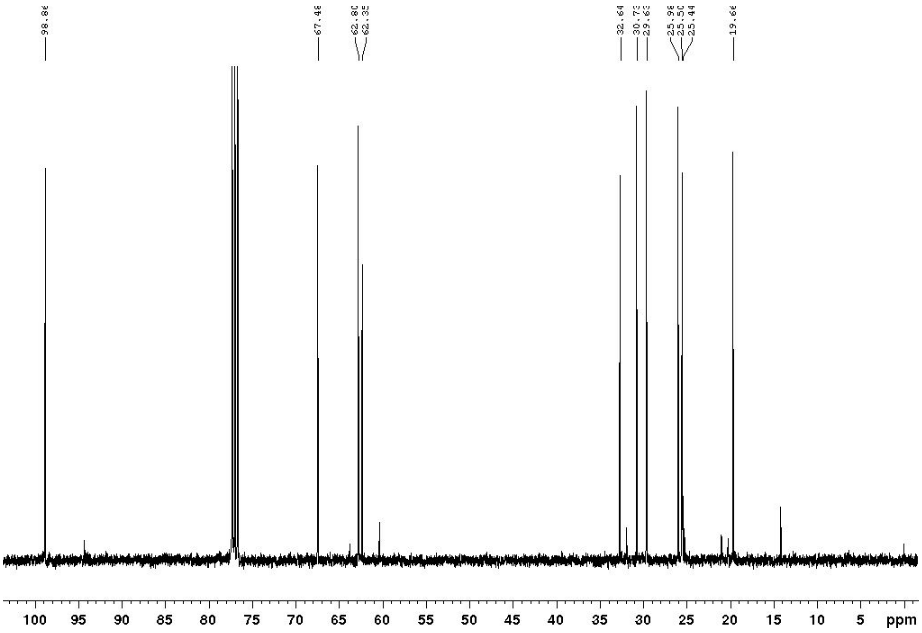

Figure S2- ^{13}C NMR spectrum ($CDCl_3$, 100 MHz)

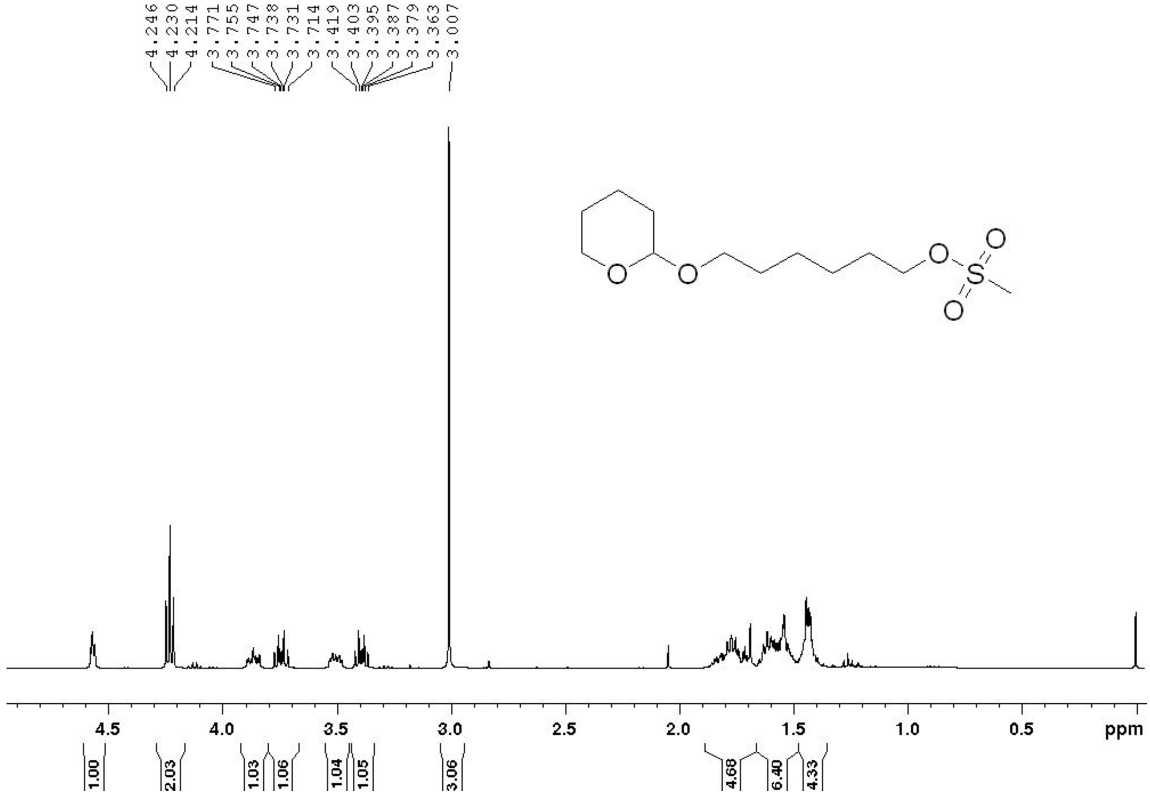

Figure S3- ^{1}H NMR spectrum ($CDCl_3$, 400 MHz)

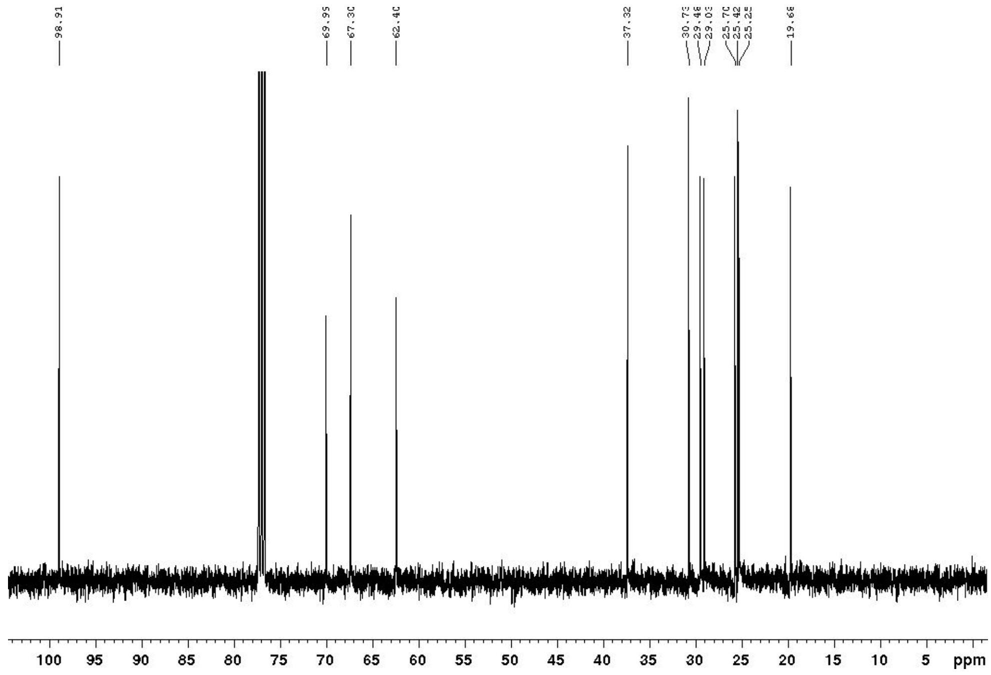

Figure S4- ^{13}C NMR spectrum ($CDCl_3$, 100 MHz)

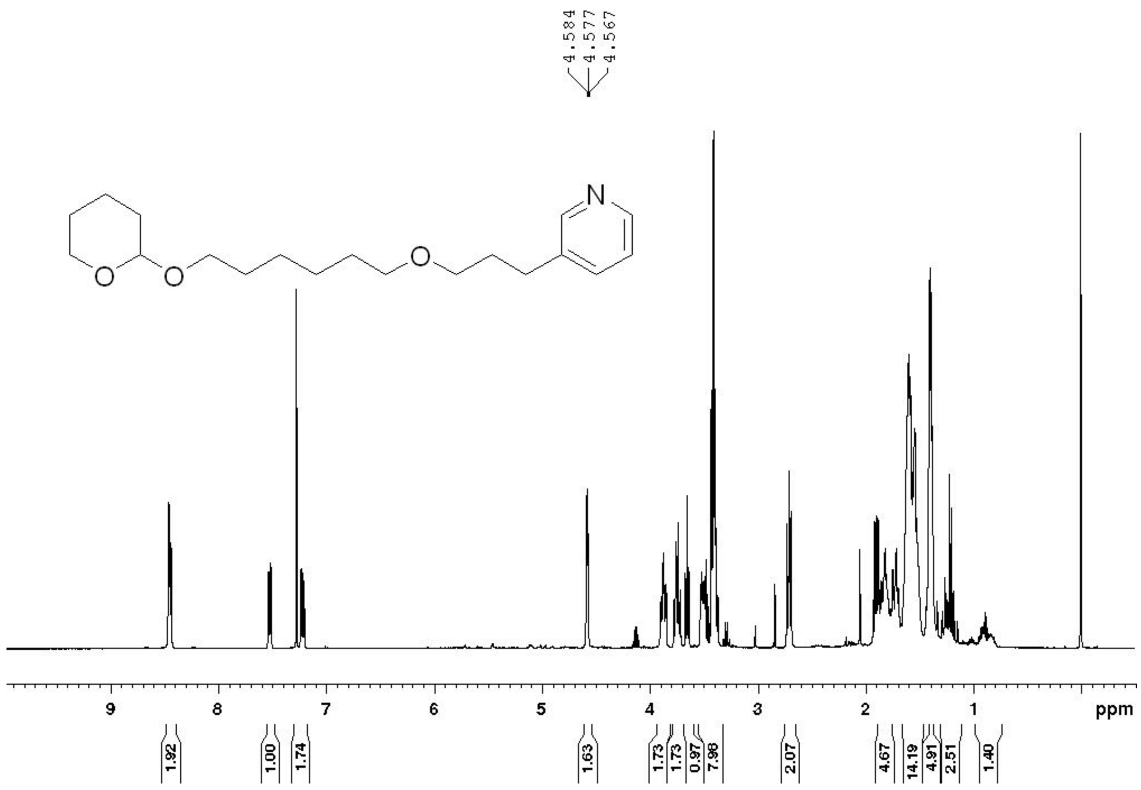

Figure S5- ^{1}H NMR spectrum ($CDCl_3$, 400 MHz)

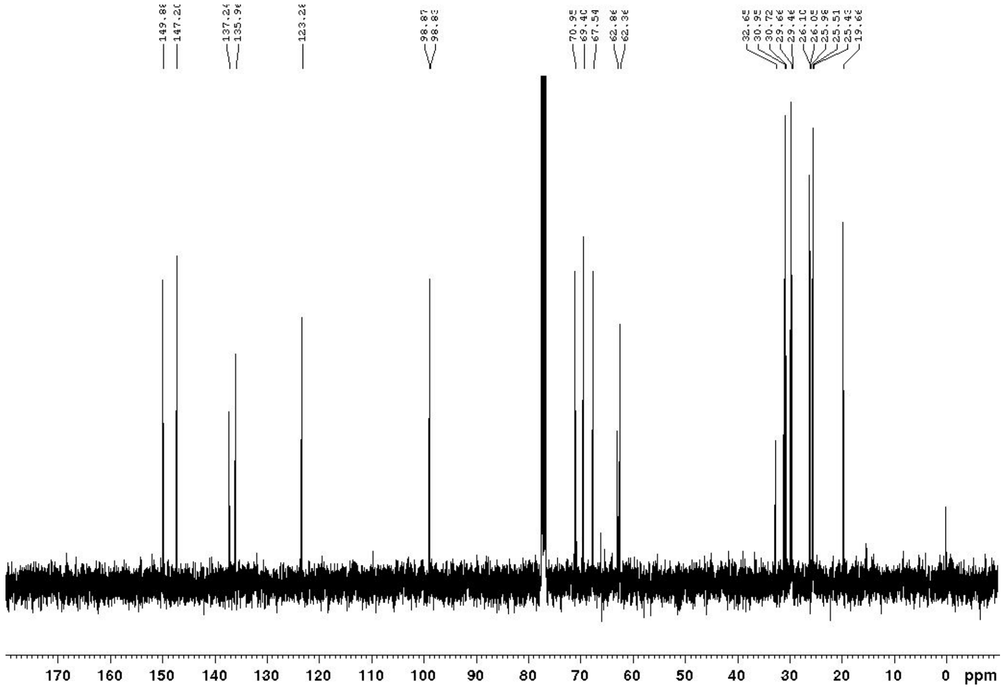

Figure S6- ^{13}C NMR spectrum ($CDCl_3$, 100 MHz)

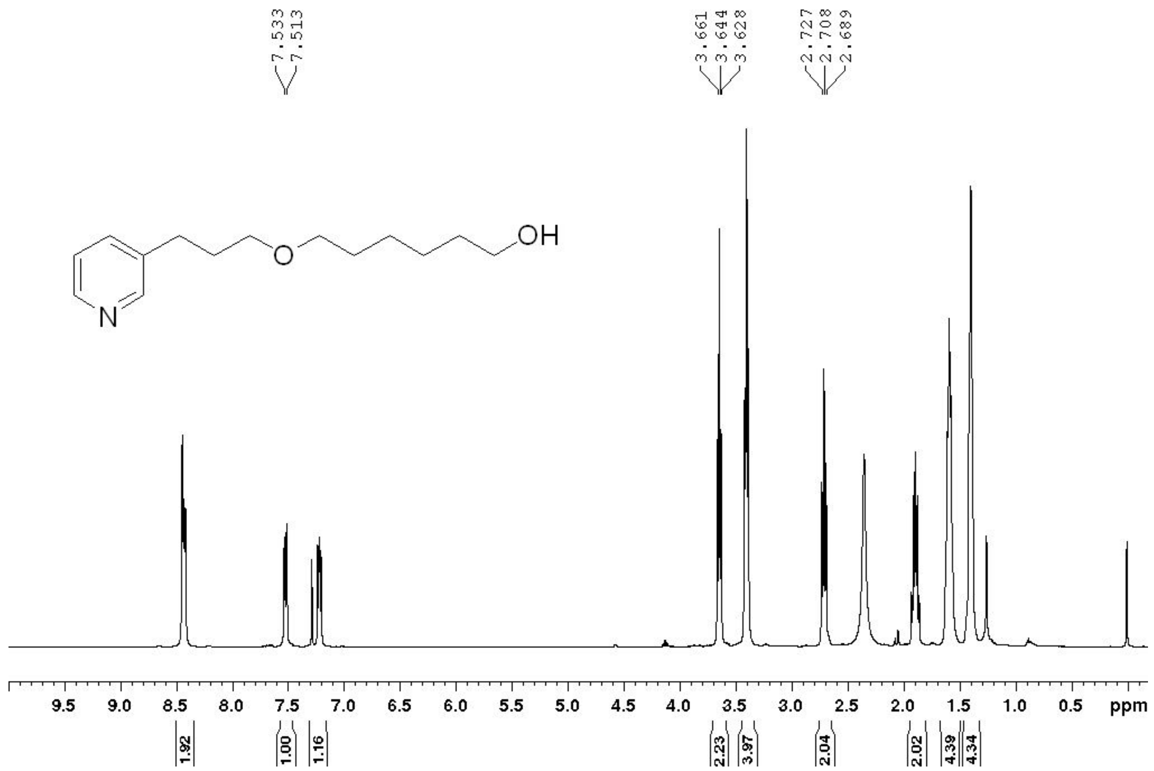

Figure S7- ^{1}H NMR spectrum ($CDCl_3$, 400 MHz)

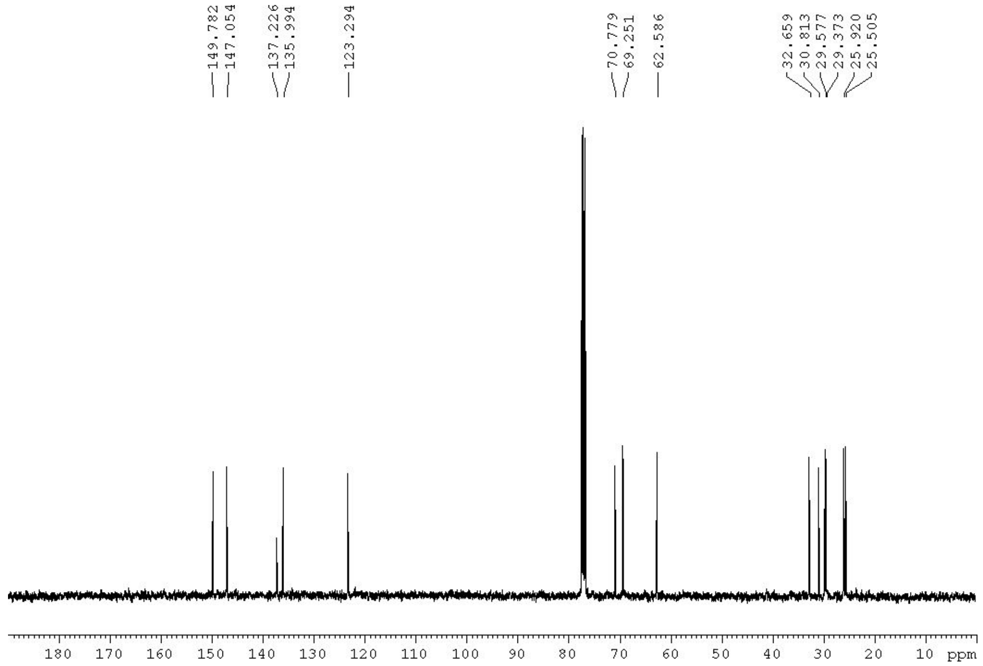

Figure S8- ^{13}C NMR spectrum ($CDCl_3$, 100 MHz)

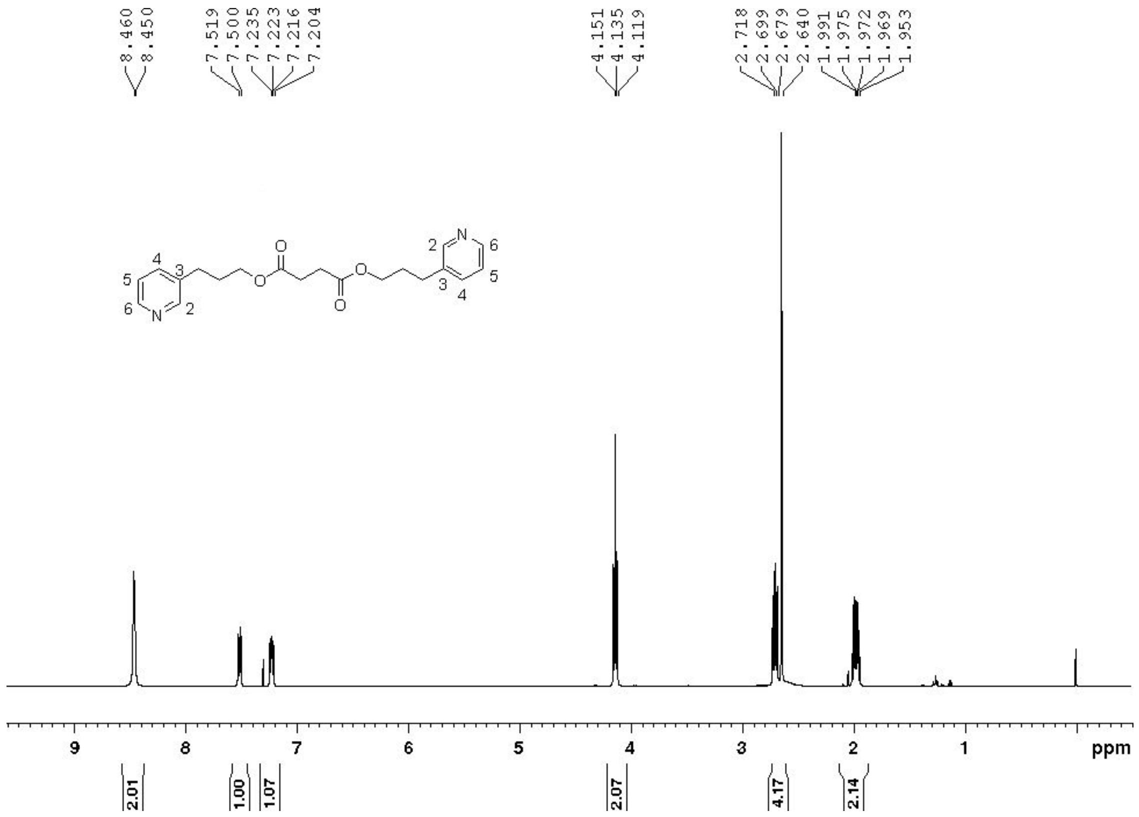

Figure S9- ^{1}H NMR spectrum ($CDCl_3$, 400 MHz)

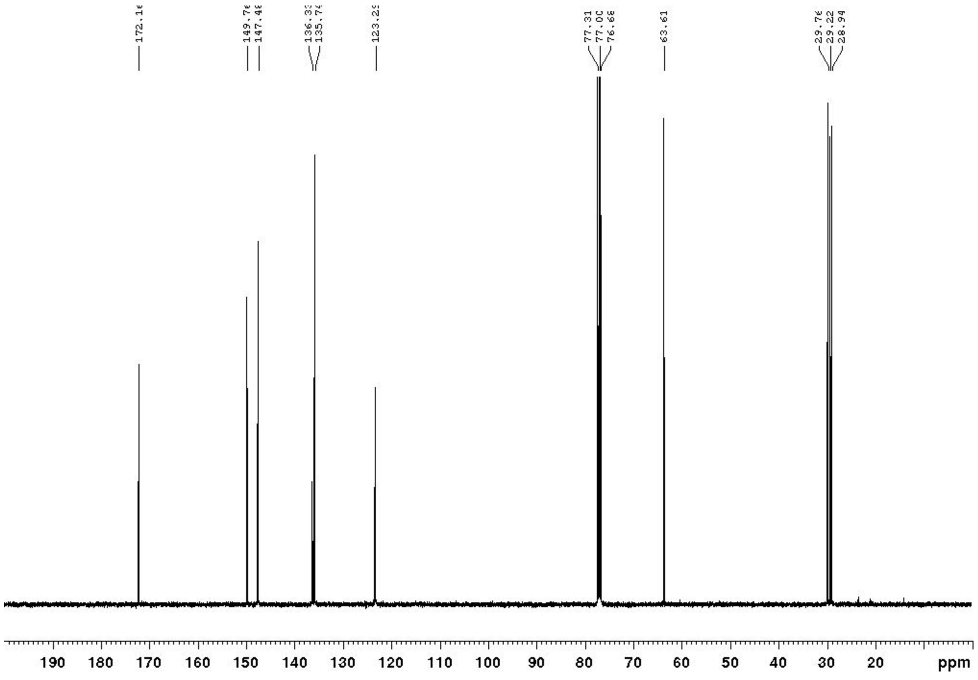

Figure S10- ^{13}C NMR spectrum ($CDCl_3$, 100 MHz)

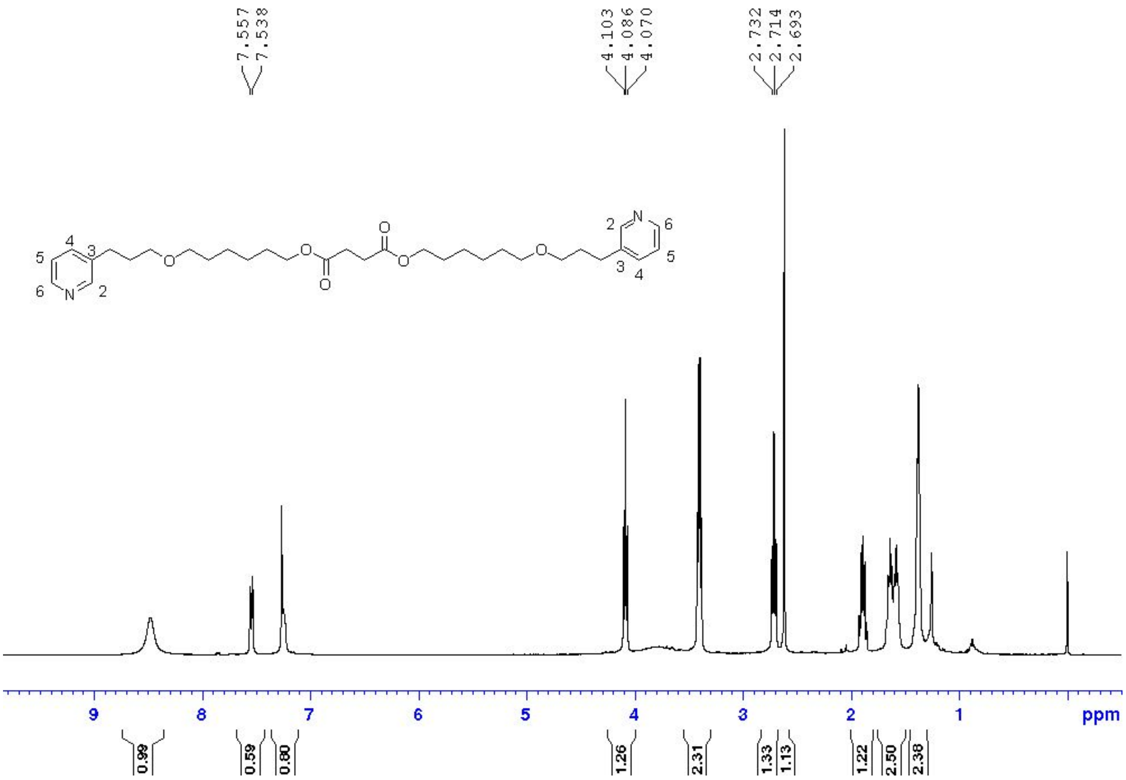

Figure S11- ^{1}H NMR spectrum ($CDCl_3$, 400 MHz)

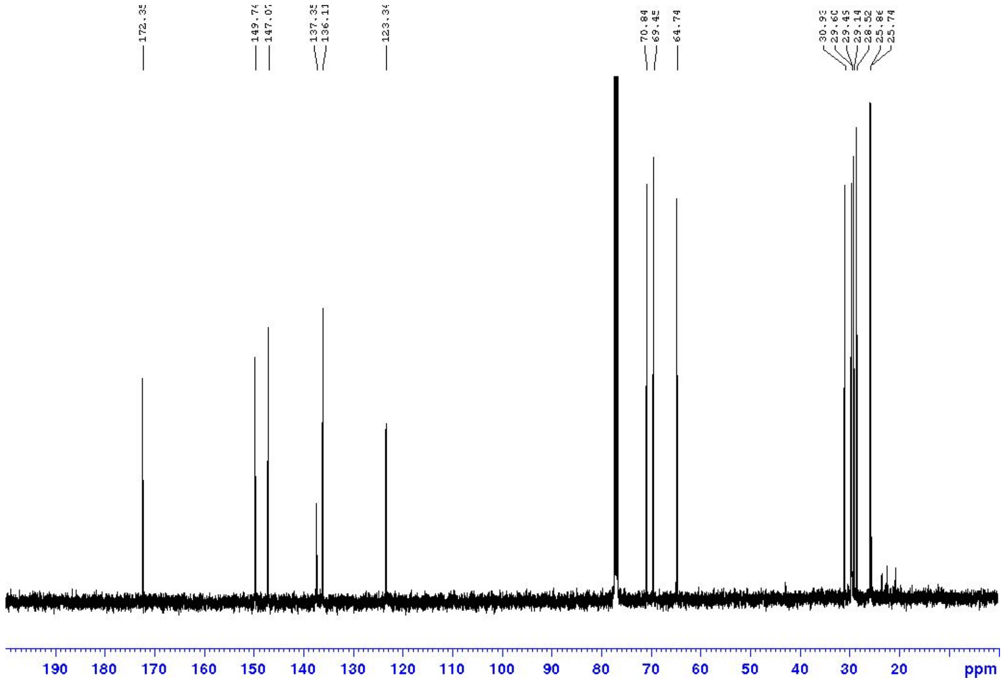

Figure S12- ^{13}C NMR spectrum ($CDCl_3$, 100 MHz)

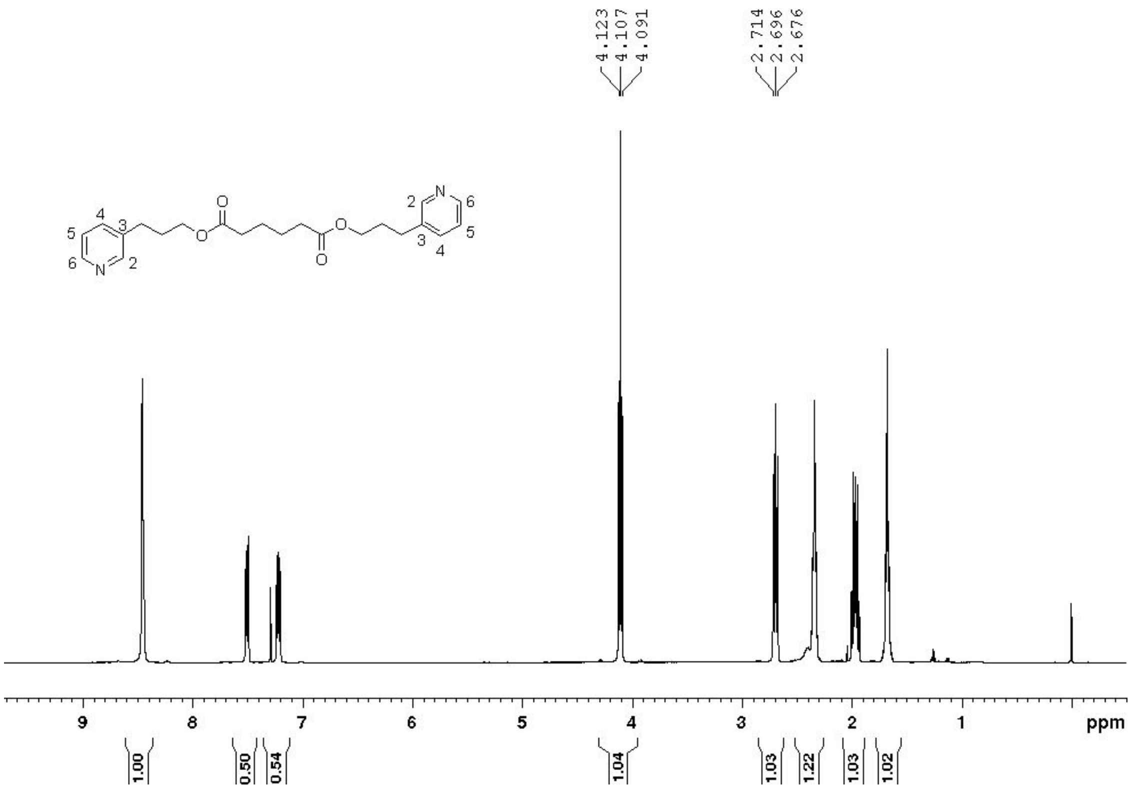

Figure S13- ^{1}H NMR spectrum ($CDCl_3$, 400 MHz)

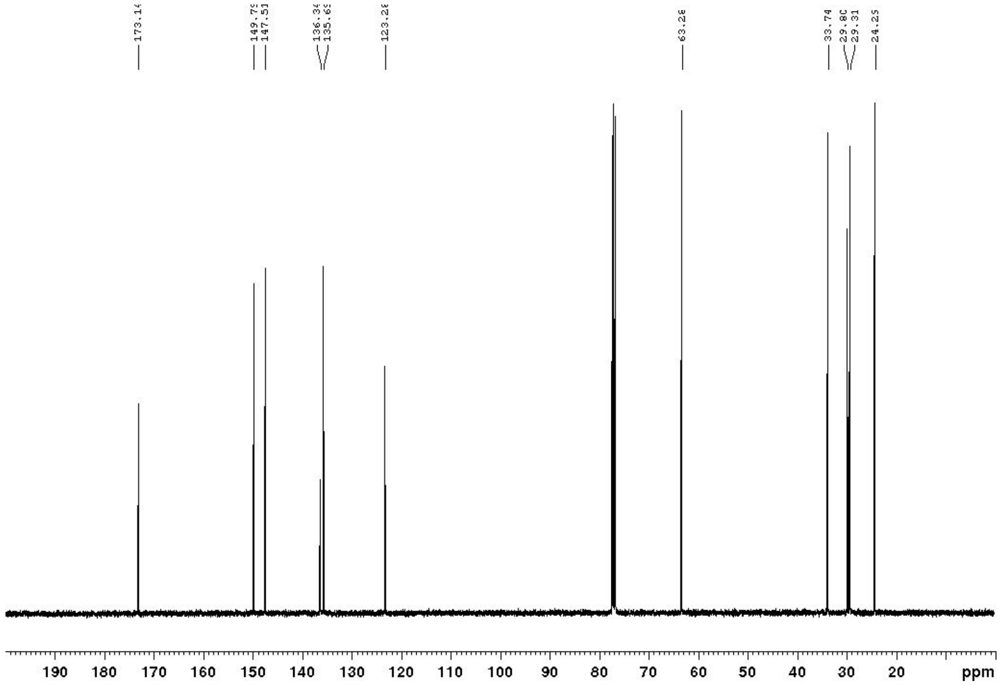

Figure S14- ^{13}C NMR spectrum ($CDCl_3$, 100 MHz)

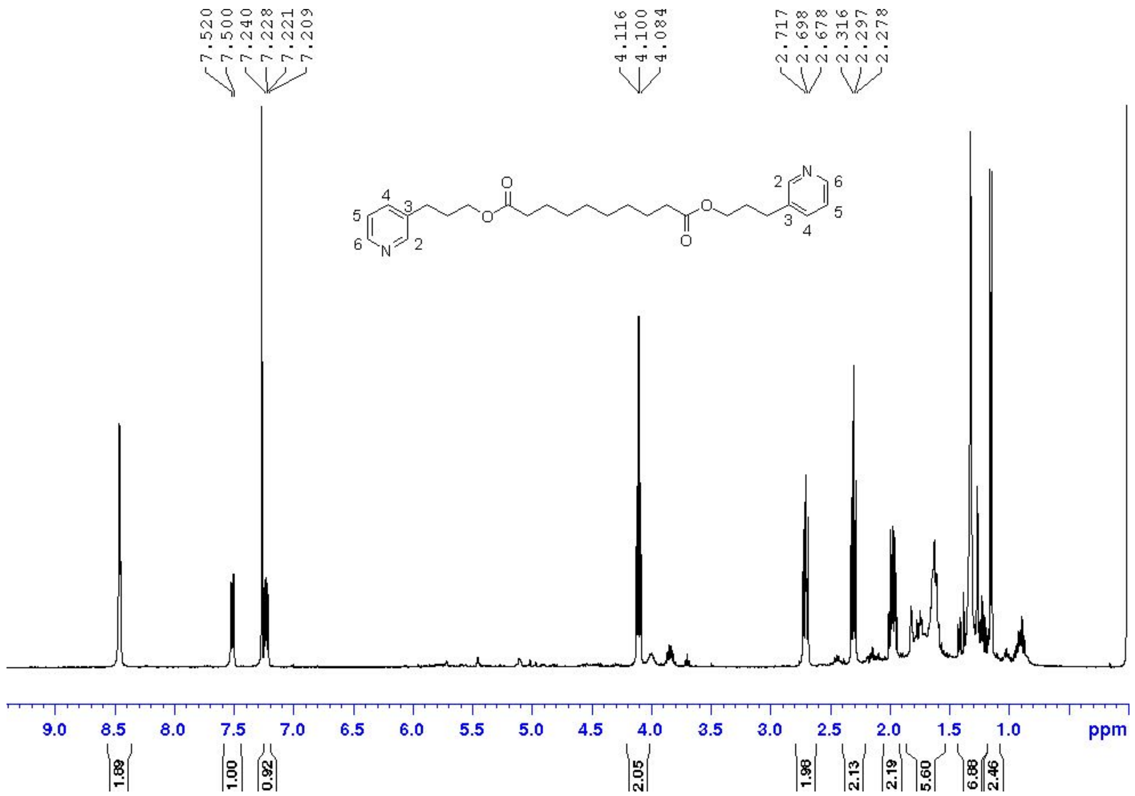

Figure S15- ^{1}H NMR spectrum ($CDCl_3$, 400 MHz)

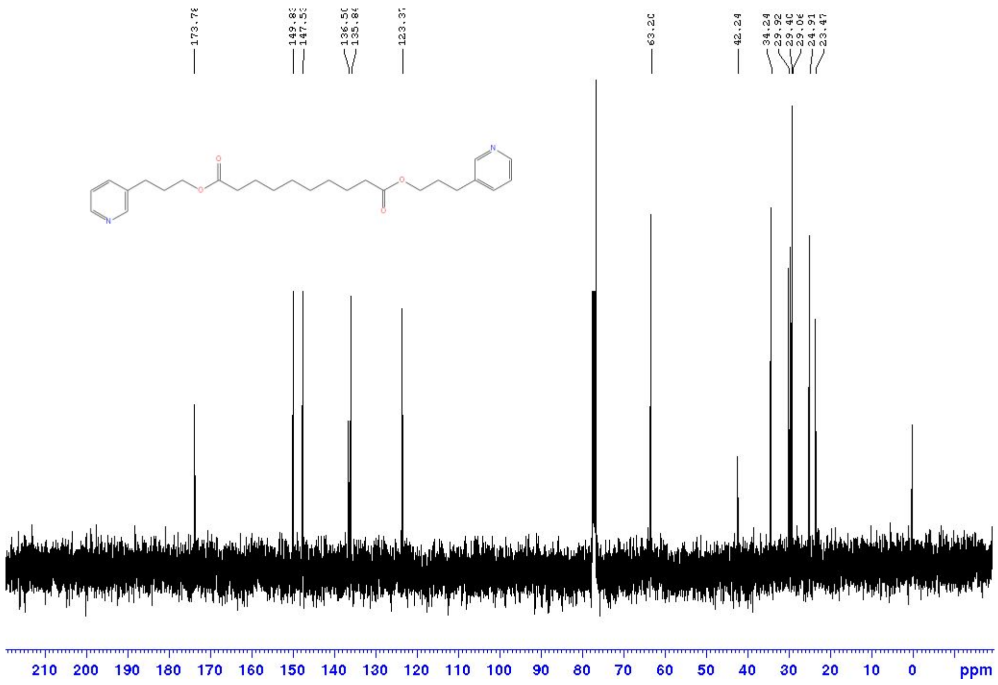

Figure S16- ^{13}C NMR spectrum ($CDCl_3$, 100 MHz)

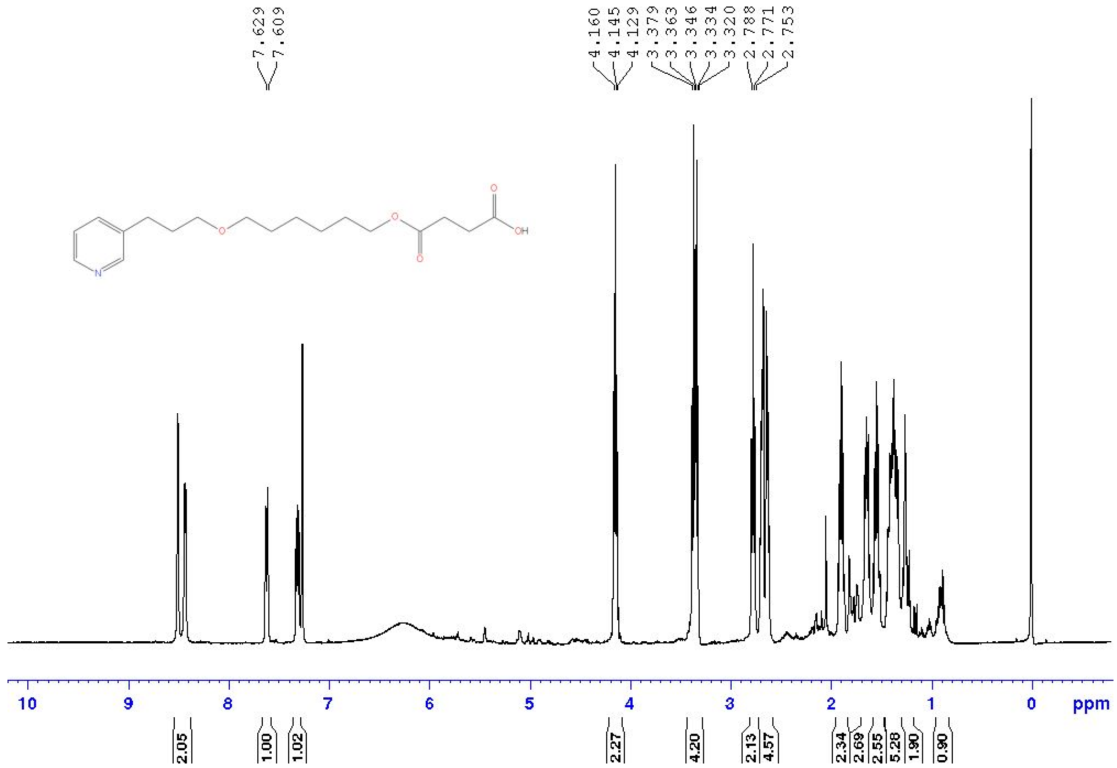

Figure S17- ¹H NMR spectrum ($CDCl_3$, 400 MHz)

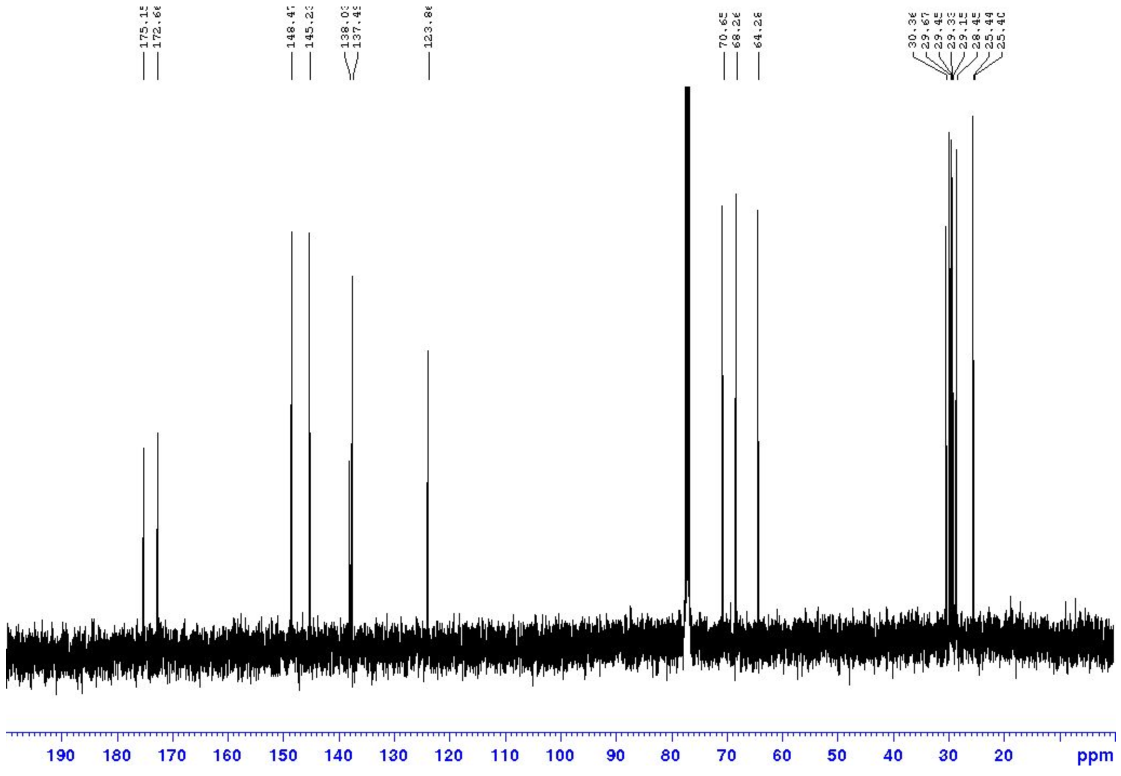

Figure S18- ¹³C NMR spectrum ($CDCl_3$, 100 MHz)

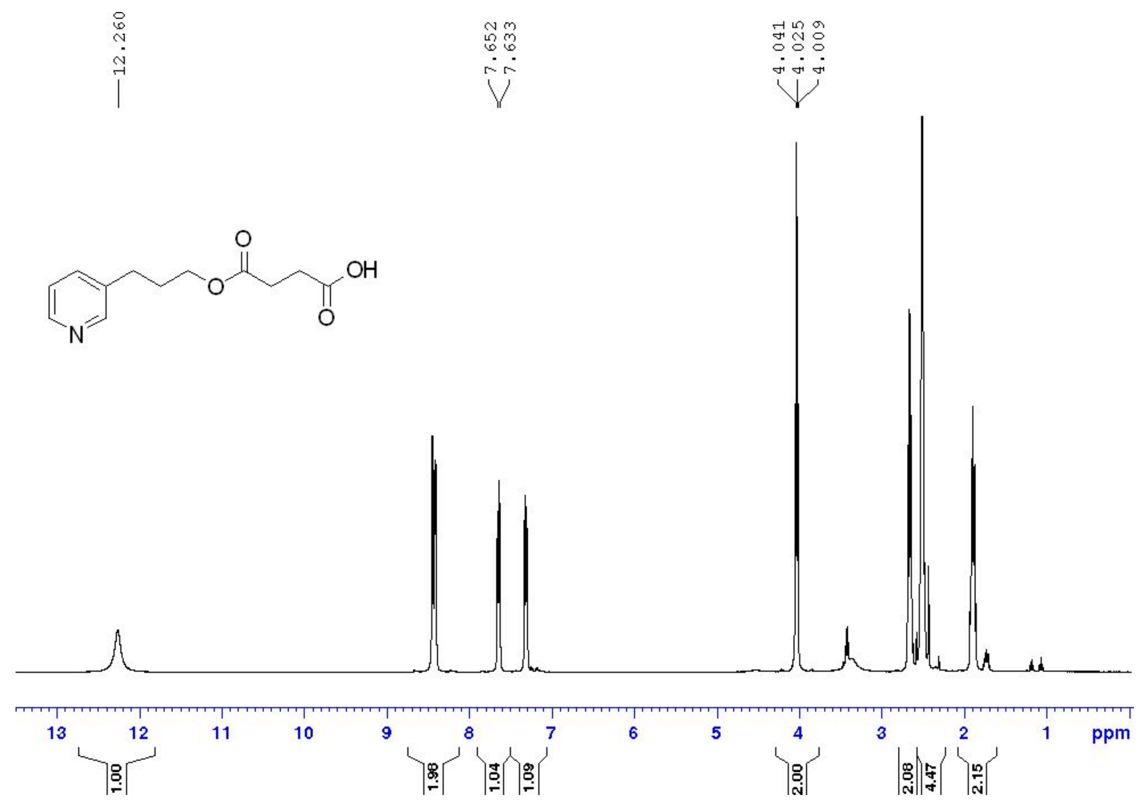

Figure S19- ^{1}H NMR spectrum ($CDCl_3$, 400 MHz)

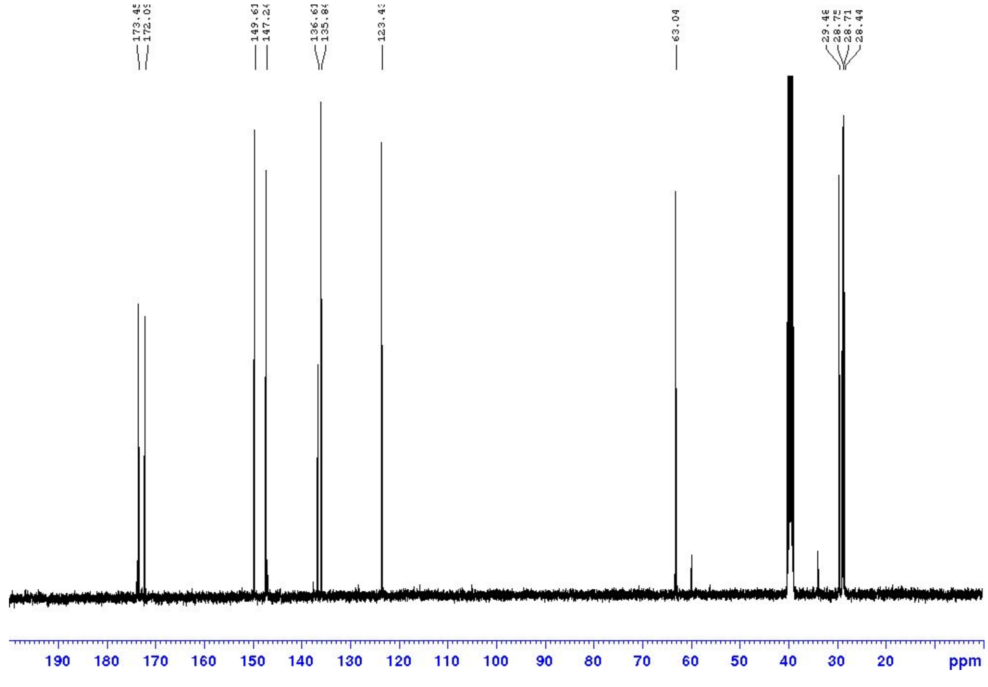

Figure S20- ^{13}C NMR spectrum ($CDCl_3$, 100 MHz)
